# Clinical outcomes of T-cell acute lymphoblastic leukemia versus T-cell lymphoblastic lymphoma after allogeneic hematopoietic stem cell transplantation

**DOI:** 10.1007/s00277-026-07295-4

**Published:** 2026-09-29

**Authors:** Jiayuan Guo, Liuqing Huang, Zixuan Li, Andi Zhang, Meng Li, Linlin Zhang, Lu Wang, Fei Li, Lili Wang, Yu Jing, Jian Bo, Daihong Liu, Liping Dou, Zhenyang Gu, Chunji Gao

**Affiliations:** 1 School of Medicine, Nankai University, Tianjin, China; 2 Medical School of Chinese PLA, Beijing, China; 3 Senior Department of Hematology, the Fifth Medical Center of PLA General Hospital, Beijing, China

**Keywords:** T-cell acute lymphoblastic leukemia, T-cell lymphoblastic lymphoma, Allogeneic stem cell transplantation, Propensity score matching

## Abstract

Although T-cell acute lymphoblastic leukemia (T-ALL) and T-cell lymphoblastic lymphoma (T-LBL) were historically regarded as different manifestations of a single disease entity driven by immature T-lymphoblast proliferation, recent advances in molecular diagnostics and cytogenetic testing have revealed inherent differences in their molecular signatures and chromosomal profiles. Despite these differences, comparative data on clinical outcomes following allogeneic hematopoietic stem cell transplantation (allo-HSCT) between the two subtypes remain limited. To address this gap, we retrospectively analyzed 106 consecutive patients, including 71 with T-ALL and 35 with T-LBL, who underwent allo-HSCT at our institution between January 2010 and January 2025. No statistically significant differences were observed in overall survival (OS), disease-free survival (DFS), graft-versus-host disease–free, relapse-free survival (GRFS), cumulative incidence of relapse (CIR), or cumulative incidence of non-relapse mortality (NRM) between patients with T-ALL and T-LBL. Diagnosis was not associated with these outcomes in multivariable analyses. However, the association between diagnosis and OS, DFS, CIR, and NRM varied over time. At 6 months, the multivariable analysis showed worse OS in T-LBL (hazard ratio at the specified time [HR(t)], 2.41; 95% confidence interval [CI], 1.10–5.28; *P* = 0.028). At 36 months, T-LBL was associated with lower risks of DFS failure (HR(t), 0.16; 95% CI, 0.03–0.91; *P* = 0.039) and NRM (subdistribution hazard ratio at the specified time [sHR(t)], 0.06; 95% CI, 0.01–0.72; *P* = 0.026). The 36-month restricted mean survival time (RMST) for OS was 5.79 months shorter in T-LBL (95% CI, − 11.38 to − 0.19; *P* = 0.043), whereas DFS and GRFS RMST did not differ significantly. Results for most outcomes did not differ significantly between groups in either the PSM cohorts or the first complete remission subgroup, although moderate to severe chronic graft-versus-host disease (cGVHD) was less frequent in T-LBL. Complete case analyses for white blood cell (WBC) and lactic dehydrogenase (LDH) and analyses restricted to patients in complete remission with evaluable measurable residual disease (MRD) did not materially alter the diagnosis estimates. Overall, OS, DFS, CIR, NRM, and GRFS did not differ significantly between patients with T-ALL and T-LBL, although the effects of diagnosis on OS, DFS, CIR, and NRM changed during follow-up. These findings should be interpreted cautiously and confirmed in larger multicenter studies.

**Supplementary Information:**

The online version contains supplementary material available at https://doi.org/10.1007/s00277-026-07295-4.

## Introduction

T-cell lymphoblastic lymphoma (T-LBL) and T-cell acute lymphoblastic leukemia (T-ALL) are hematological malignancies that originate from immature T cells. These malignancies mainly involve the mediastinum and other lymphoid organs, and may spread to the peripheral blood, bone marrow, and central nervous system (CNS) [1, 2]. Historically, based on clinical, immunophenotypic and molecular characteristics, studies have considered T-ALL and T-LBL as distinct manifestations of the same disease [3, 4]. However, mounting evidence now suggests that there are distinct differences in the molecular biological and cytogenetic features between T-LBL and T-ALL, necessitating a differentiated therapeutic approach to prolong survival and improve quality of life [5–11].

Allogeneic hematopoietic stem cell transplantation (allo-HSCT) leverages the immune-mediated graft-versus-leukemia effect to eradicate leukemic cells, making it a promising therapeutic approach for T-ALL and T-LBL [12–14]. Moreover, it has demonstrated efficacy in T-ALL and T-LBL patients, regardless of whether they were in complete remission (CR) or had relapsed/refractory disease prior to allo-HSCT [14–19]. Due to the limited data available, whether allo-HSCT provides similar benefit in T-ALL and T-LBL remains unclear. Consequently, there is a critical need to investigate whether the underlying biological differences between T-ALL and T-LBL could result in diverse clinical outcomes following allo-HSCT. Therefore, our retrospective study aimed to compare posttransplant outcomes between patients with T-ALL and T-LBL who underwent allo-HSCT.

## Methods

### Patients

Between January 2010 and January 2025, patients with T-ALL or T-LBL who received allogeneic hematopoietic cell transplantation following myeloablative conditioning were retrospectively reviewed at the Department of Hematology, the First Medical Center of Chinese PLA General Hospital. The diagnosis of T-ALL and T-LBL was retrospectively reviewed with reference to the fifth edition of the World Health Organization Classification of Haematolymphoid Tumours (WHO-HAEM5) and the 2022 International Consensus Classification (ICC), integrating morphologic and immunophenotypic findings with cytogenetic and molecular findings when performed [20, 21]. T-LBL was defined by predominant nodal or extranodal disease with bone marrow lymphoblasts < 25%, whereas T-ALL was defined by ≥ 25% bone marrow lymphoblasts. To maintain consistency with previously published transplantation studies in similar disease settings [22, 23], the study cohort was restricted to patients aged ≥ 16 years at the time of transplantation. All participants underwent standard chemotherapy protocols prior to allo-HSCT. Exclusion criteria included bi-phenotypic leukemia, mixed-lineage acute lymphoblastic leukemia, human T-lymphotropic virus (HTLV)-positive adult T-cell leukemia/lymphoma, and receipt of reduced-intensity conditioning regimens. In addition, patients with insufficient baseline information to confirm study eligibility or define the core study variables were also excluded. Data on patient demographics and treatment modalities were systematically collected from electronic medical records. The procedures adhered to the tenets of the Declaration of Helsinki. The requirement for informed consent was waived.

### Transplantation procedure

All eligible patients were advised to undergo allo-HSCT in accordance with institutional practice, regardless of their CR status. This was particularly emphasized for those in second complete remission (CR2) or higher, as well as patients in first complete remission (CR1) who exhibited one or more traditional high-risk features, including: high white blood cell count (WBC > 100 × 10^9^/L), CNS involvement at diagnosis, age ≥ 35 years, the requirement for multiple cycles of induction therapy to achieve CR1, the detection of measurable residual disease (MRD) by flow cytometry, or the early T-cell precursor (ETP) subtype [24]. Donor selection followed protocols established by the Chinese Society of Hematology [25]. Peripheral blood stem cells, mobilized using recombinant human granulocyte colony-stimulating factor (rhG-CSF), served as the source of grafts in this study. The conditioning regimens used in this study were all myeloablative and were as described previously [26, 27]. All patients received cyclosporine (CsA), short-term methotrexate (MTX), and mycophenolate mofetil (MMF) for graft-versus-host disease (GVHD) prophylaxis. For those undergoing allo-HSCT from human leukocyte antigen (HLA)-matched sibling donors (MSD) before 2017, the regimen excluded anti-thymocyte globulin (ATG; thymoglobulin, rabbit; Genzyme Europe BV, Naarden, the Netherlands) [28, 29]. For the remaining patients, ATG was administered at a dosage of 2.5 mg/kg/day on days − 5 to -4 in MSD recipients, and on days − 5 to -2 in recipients of grafts from HLA-haploidentical related donors (HID) or HLA-matched unrelated donors (MUD) [30].

### Definitions and endpoints

The precise characterization of the ETP subtype was accomplished through comprehensive analysis of bone marrow samples using 8- to 10-color multiparameter flow cytometry, and was defined by the absence of CD1a and CD8 expression, absent or weak CD5 expression (< 75%), and the expression of at least one myeloid/stem cell marker, such as CD117, HLA-DR, CD13, CD33, CD11b, or CD65 [31]. Cytogenetic analysis was limited to marrow specimens obtained from patients. Somatic mutations were assessed using targeted capture sequencing to identify gene aberrations, as described previously [32–34].

Treatment response was assessed by bone marrow examination and computed tomography (CT) or positron emission tomography/computed tomography (PET/CT), as clinically appropriate. Response in patients with T-LBL was evaluated according to the Lugano classification. For T-ALL, CR was defined by morphologic criteria: bone marrow blasts < 5% and no evidence of extramedullary disease. Measurable residual disease (MRD) before allo-HSCT was assessed by multiparameter flow cytometry through the identification of a distinct cell population with leukemia-associated immunophenotypes (LAIPs) or abnormal antigen expression patterns relative to normal or regenerating bone marrow. Relapse or disease progression was defined from medical records based on the documented reappearance or increase of leukemic blasts in peripheral blood or bone marrow, or new or progressive extramedullary disease. Overall survival (OS) was measured from graft infusion to death from any cause or the last follow-up. Disease-free survival (DFS) was defined as the time from graft infusion to the occurrence of disease relapse, progression, or death. Non-relapse mortality (NRM) denoted death related to transplantation without relapse or disease progression and was calculated from the date of transplantation. Graft-versus-host disease–free relapse-free survival (GRFS) was measured from transplantation to the onset of grade III-IV acute GVHD, the development of chronic graft-versus-host disease (cGVHD) requiring systemic immunosuppression, leukemia relapse, or death from any cause.

Acute graft-versus-host disease (aGVHD) was diagnosed and graded using the modified Glucksberg criteria [35, 36]. Chronic GVHD was diagnosed and graded according to the 2014 National Institutes of Health (NIH) consensus criteria [37]. Moderate–severe cGVHD was defined as the first occurrence of moderate or severe cGVHD, including progression from initially mild cGVHD. Definitions for hematologic recovery included neutrophil engraftment, identified as an absolute neutrophil count exceeding 0.5 × 10^9^/L for 3 consecutive days without administration of G-CSF, and platelet engraftment, identified as an absolute platelet count surpassing 20 × 10^9^/L for 7 consecutive days without platelet transfusion [38].

### Statistical analysis

Categorical variables were summarized as counts and percentages and compared using the chi-square test or Fisher’s exact test, as appropriate. Continuous variables were presented as medians with interquartile ranges (IQRs) and compared using the Mann–Whitney U test. Transplant era was categorized as 2010–2018 and 2019–2025 based on the median year of transplantation. To reduce baseline imbalances between the T-ALL and T-LBL groups, propensity score matching (PSM) was applied [39]. Details of the matching procedure and balance assessment are provided in the Supplementary Methods.

The median follow-up duration was estimated using the reverse Kaplan–Meier method. OS, DFS, and GRFS were estimated using the Kaplan–Meier method and compared with the log-rank test. Cox regression was used to estimate hazard ratios (HRs) and 95% confidence intervals (CIs) for OS, DFS, and GRFS. The proportional hazards assumption was assessed using Schoenfeld residuals, and covariates that violated the assumption were further analyzed using an interaction with log(1 + time). Cumulative incidences of relapse, NRM, and GVHD were analyzed using competing risk methods and compared using Gray’s test. Relapse and NRM were treated as mutually competing events. For aGVHD and cGVHD, relapse or death before the event of interest was treated as a competing event. Fine–Gray regression was used to estimate subdistribution hazard ratios (sHRs) and 95% CIs for cumulative incidence of relapse (CIR), NRM, and GVHD. The proportional subdistribution hazards assumption for selected covariates was assessed using an interaction with log(1 + time). For covariates showing time-varying effects, sHRs were estimated at selected time points. Details of the regression and time interaction analyses are provided in the Supplementary Methods.

Missing values were not imputed. To assess the influence of missing WBC and lactic dehydrogenase (LDH) data, multivariable analyses for OS, DFS, and GRFS were performed among patients with recorded WBC and/or LDH values, both before and after adding WBC, LDH, or both as covariates. CIR was similarly examined by adding LDH among patients with recorded LDH. The associations of diagnosis and pretransplant MRD with OS and DFS were examined among patients in CR with evaluable MRD. Further details are provided in the Supplementary Methods.

The 36-month restricted mean survival time (RMST) was estimated for OS, DFS, and GRFS. The 36-month restricted mean time lost (RMTL) was estimated for CIR, NRM, any NIH cGVHD, and moderate to severe cGVHD. The 100-day RMTL was estimated for grade II–IV and grade III–IV aGVHD. Details of the restricted mean analyses are provided in the Supplementary Methods.

All analyses were performed using SPSS version 27.0.1 (IBM, Armonk, NY) and R version 4.2.3, with two-sided P values < 0.05 considered statistically significant.

## Results

### Patients and allo-HSCT characteristics

A total of 113 consecutive patients with T-ALL or T-LBL who underwent allo-HSCT between January 2010 and January 2025 were retrospectively reviewed. Seven patients were excluded. Six were younger than 16 years, and one had no recorded bone marrow blast percentage at diagnosis. Ultimately, data from 106 patients were included in the final analysis (Table 1). Of these, 71 patients were diagnosed with T-ALL, and 35 with T-LBL. At diagnosis, patients with T-ALL had a significantly higher bone marrow blast burden than those with T-LBL (median 82.4% vs. 1.2%, *P* < 0.001). A higher proportion of patients with WBC > 100 × 10^9^/L was observed in T-ALL compared with T-LBL, although the difference was not statistically significant (11.3% vs. 0%, *P* = 0.051). At the time of allo-HSCT, a greater proportion of patients with T-ALL were in CR1 compared with those with T-LBL (87.3% vs. 68.6%, *P* = 0.028). Other baseline characteristics were comparable between the two groups. WBC was unavailable in 2 patients (1.9%), and LDH was unavailable in 14 patients (13.2%). Missing data and their analytical handling are summarized in Table S1.

**Table 1:** Clinical characteristics of all patients

Characteristic	Total (*n* = 106)	T-ALL (*n* = 71)	T-LBL (*n* = 35)	*P* value
Age at allo-HSCT, years				
Median (IQR)	25.0 (20.2–32.0)	26.0 (20.0-32.5)	24.0 (21.0–32.0)	0.851
≤ 35	87 (82.1%)	58 (81.7%)	29 (82.9%)	0.883
> 35	19 (17.9%)	13 (18.3%)	6 (17.1%)	
Sex, n (%)				0.678
Female	30 (28.3%)	21 (29.6%)	9 (25.7%)	
Male	76 (71.7%)	50 (70.4%)	26 (74.3%)	
BM blasts at diagnosis, %, median (IQR)	58.0 (9.5–88.5)	82.4 (58.0–93.0)	1.2 (0.4–8.6)	< 0.001
WBC at diagnosis, n (%)				0.051
≤ 100 × 10^9/L	96 (90.6%)	62 (87.3%)	34 (97.1%)	
> 100 × 10^9/L	8 (7.5%)	8 (11.3%)	0 (0.0%)	
Missing	2 (1.9%)	1 (1.4%)	1 (2.9%)	
LDH at diagnosis, n (%)				0.953
Normal	44 (41.5%)	30 (42.3%)	14 (40.0%)	
Elevated	48 (45.3%)	33 (46.5%)	15 (42.9%)	
Missing	14 (13.2%)	8 (11.3%)	6 (17.1%)	
CNS involvement at diagnosis, n (%)				0.549
No	103 (97.2%)	68 (95.8%)	35 (100.0%)	
Yes	3 (2.8%)	3 (4.2%)	0 (0.0%)	
Mediastinal involvement at diagnosis, n (%)				0.088
No	64 (60.4%)	46 (64.8%)	18 (51.4%)	
Yes	38 (35.8%)	21 (29.6%)	17 (48.6%)	
Missing	4 (3.8%)	4 (5.6%)	0 (0.0%)	
ETP immunophenotype at diagnosis, n (%)				0.050
Non-ETP	62 (58.5%)	37 (52.1%)	25 (71.4%)	
ETP	34 (32.1%)	27 (38.0%)	7 (20.0%)	
Missing	10 (9.4%)	7 (9.9%)	3 (8.6%)	
HCT-CI, n (%)				0.213
0	78 (73.6%)	56 (78.9%)	22 (62.9%)	
1	22 (20.8%)	12 (16.9%)	10 (28.6%)	
≥2	6 (5.7%)	3 (4.2%)	3 (8.6%)	
Disease status at allo-HSCT, n (%)				0.028
CR1	86 (81.1%)	62 (87.3%)	24 (68.6%)	
CR2	8 (7.5%)	5 (7.0%)	3 (8.6%)	
Non-CR	12 (11.3%)	4 (5.6%)	8 (22.9%)	
MRD status before allo-HSCT, n (%)				0.137
MRD−	77 (81.9%)	52 (77.6%)	25 (92.6%)	
MRD+	17 (18.1%)	15 (22.4%)	2 (7.4%)	
Cycles of chemotherapy before allo-HSCT				
Median (IQR)	5.0 (4.0–6.0)	5.0 (4.0–6.0)	5.0 (4.0-6.5)	0.180
≤4	36 (34.0%)	26 (36.6%)	10 (28.6%)	0.411
≥5	70 (66.0%)	45 (63.4%)	25 (71.4%)	
Time from diagnosis to allo-HSCT, months				
Median (IQR)	7.0 (6.0–9.0)	7.0 (6.0–9.0)	7.0 (6.0–9.0)	0.526
<8	59 (55.7%)	40 (56.3%)	19 (54.3%)	0.841
≥8	47 (44.3%)	31 (43.7%)	16 (45.7%)	
Year of transplantation				
Median (IQR)	2018 (2016–2020)	2019 (2016–2020)	2017 (2016–2020)	0.209
2010–2018	53 (50.0%)	32 (45.1%)	21 (60.0%)	0.148
2019–2025	53 (50.0%)	39 (54.9%)	14 (40.0%)	
Conditioning regimen, n (%)				0.419
mBuCy	79 (74.5%)	52 (73.2%)	27 (77.1%)	
TBI-Cy	21 (19.8%)	16 (22.5%)	5 (14.3%)	
Others	6 (5.7%)	3 (4.2%)	3 (8.6%)	
Donor type, n (%)				0.358
MSD	29 (27.4%)	22 (31.0%)	7 (20.0%)	
HID	64 (60.4%)	42 (59.2%)	22 (62.9%)	
MUD	13 (12.3%)	7 (9.9%)	6 (17.1%)	
GVHD prophylaxis, n (%)				0.459
CSA + MTX+MMF	16 (15.1%)	12 (16.9%)	4 (11.4%)	
CSA + MTX+MMF + ATG	90 (84.9%)	59 (83.1%)	31 (88.6%)	
Donor-recipient gender matching, n (%)				0.248
Female-male	32 (30.2%)	24 (33.8%)	8 (22.9%)	
Others	74 (69.8%)	47 (66.2%)	27 (77.1%)	
MNCs × 10^8^/kg, median (IQR)	9.3 (7.7–12.7)	9.5 (7.9–13.1)	8.4 (7.3–12.5)	0.426
CD34 + cells × 10^6^/kg, median (IQR)	4.6 (3.3-6.0)	4.6 (3.5-6.0)	4.4 (3.1–5.6)	0.493

MRD status was evaluated only in patients who were in complete remission at the time of allo-HSCT, and percentages were calculated using these evaluable patients as the denominator

*T-ALL* T-cell acute lymphoblastic leukemia, *T-LBL* T-cell lymphoblastic lymphoma, *allo-HSCT*, allogeneic hematopoietic stem cell transplantation, *IQR *interquartile range, *BM *bone marrow, *WBC *white blood cell, *LDH *lactic dehydrogenase, *CNS *central nervous system, *ETP *early T-cell precursor, *HCT-CI *hematopoietic cell transplantation comorbidity index, *CR1 *first complete remission, *CR2 *second complete remission, *non-CR *non-complete remission, *MRD *measurable residual disease, *mBuCy *modified busulfan plus cyclophosphamide, *TBI-Cy *total body irradiation plus cyclophosphamide, *MSD *human leukocyte antigen (HLA)-matched sibling donors, *HID *HLA-haploidentical related donors, *MUD *HLA-matched unrelated donors, *GVHD *graft-versus-host disease, *CSA *cyclosporine A, *MMF *mycophenolate mofetil, *MTX *methotrexate, *ATG *antithymocyte globulin, *MNCs *mononuclear cells

Among patients with sufficient records to determine the relevant intrathecal therapy status, prophylactic therapy was administered before and after allo-HSCT in 92 of 99 patients (92.9%) and 10 of 74 patients (13.5%), respectively, and intrathecal therapy for documented CNS involvement was administered before and after allo-HSCT in 4 of 68 patients (5.9%) and 5 of 73 patients (6.8%), respectively. Further details are provided in Tables S2 and S3.

**Fig. 1:**
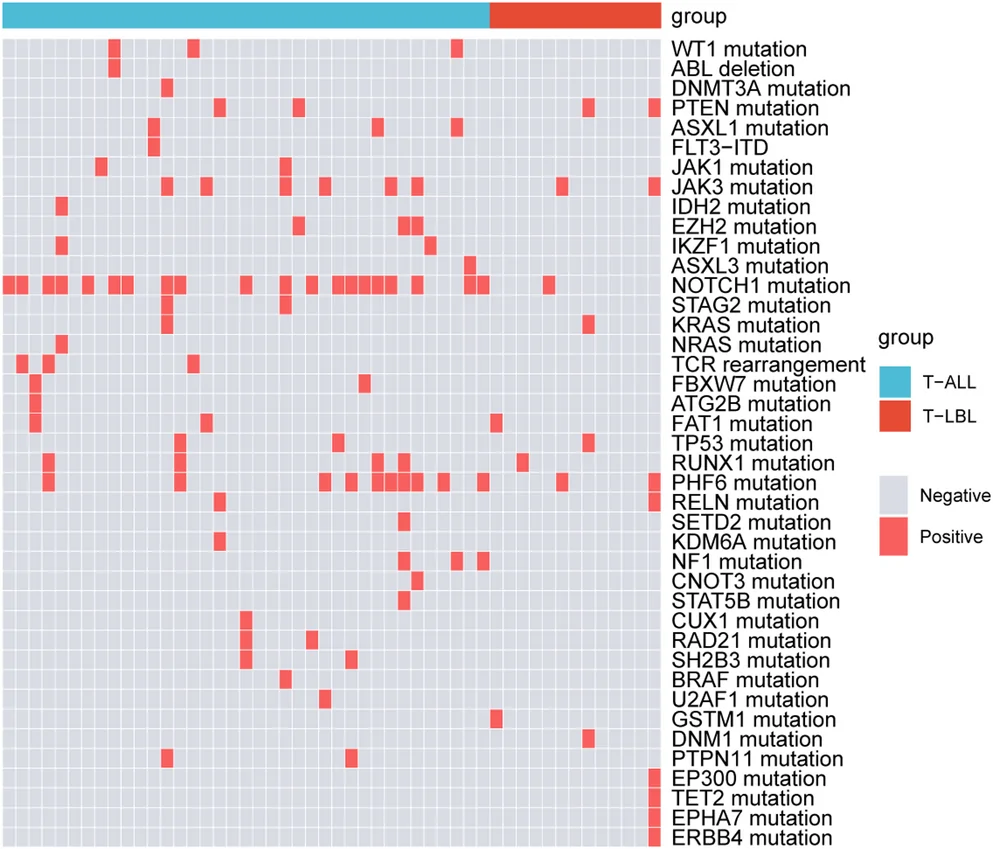
Landscape of gene aberrations in 37 patients with T-ALL and 13 patients with T-LBL. Each column represents an individual patient, and each row corresponds to a specific gene aberration. The panel is colored red if the aberration is present and gray if the gene is of the wild type. ITD, internal tandem duplication; T-ALL, T-cell acute lymphoblastic leukemia; T-LBL, T-cell lymphoblastic lymphoma

Genetic aberration data were also obtained from bone marrow samples of 37 T-ALL patients and 13 T-LBL patients (Fig. 1). No aberration among the genes shown was detected in five patients with T-ALL and seven patients with T-LBL. Among T-ALL patients, the most common genetic aberrations were identified as follows: NOTCH1^mut^, PHF6^mut^, JAK3^mut^ and RUNX1^mut^. In T-LBL patients, genetic aberrations were infrequent and heterogeneous. Genetic aberrations, such as RAD21^mut^, ASXL3^mut^, ATG2B^mut^, and ABL^del^ in T-ALL patients, as well as DNM1^mut^ and GSTM1^mut^ in T-LBL patients, were also identified in our patient cohort, despite the lack of prior reports associating these mutations with T-ALL or T-LBL. Genetic analyses were considered exploratory because genetic testing was not uniformly available throughout the study period.

### Transplantation outcomes and survival in the overall cohort

Neutrophil engraftment was achieved in both groups following allo-HSCT, with a median time to engraftment of 13 (IQR, 11–15) days for T-ALL patients and 12 (IQR, 10-14.5) days for T-LBL patients (*P* = 0.377). Platelet engraftment failure occurred in 3 T-ALL patients and 2 T-LBL patients. The median time to platelet engraftment was 13 (IQR, 11–20) days for T-ALL patients and 15 (IQR, 12–18) days for T-LBL patients (*P* = 0.744).

Using the reverse Kaplan–Meier method, the median follow-up duration was 67.5 months (95% CI, 56.5–85.9 months) for the entire cohort, 67.5 months (95% CI, 54.2–83.8 months) for the T-ALL group, and 77.8 months (95% CI, 50.0–142.3 months) for the T-LBL group. By the end of the follow-up, 36 patients (34.0%) relapsed and 51 patients (48.1%) died. Disease progression accounted for the largest proportion of deaths in both groups (T-ALL: 20/31, 64.5%; T-LBL: 11/20, 55.0%), followed by infection (T-ALL: 7/31, 22.6%; T-LBL: 3/20, 15.0%) and hemorrhage (T-ALL: 2/31, 6.5%; T-LBL: 3/20, 15.0%).

**Fig. 2:**
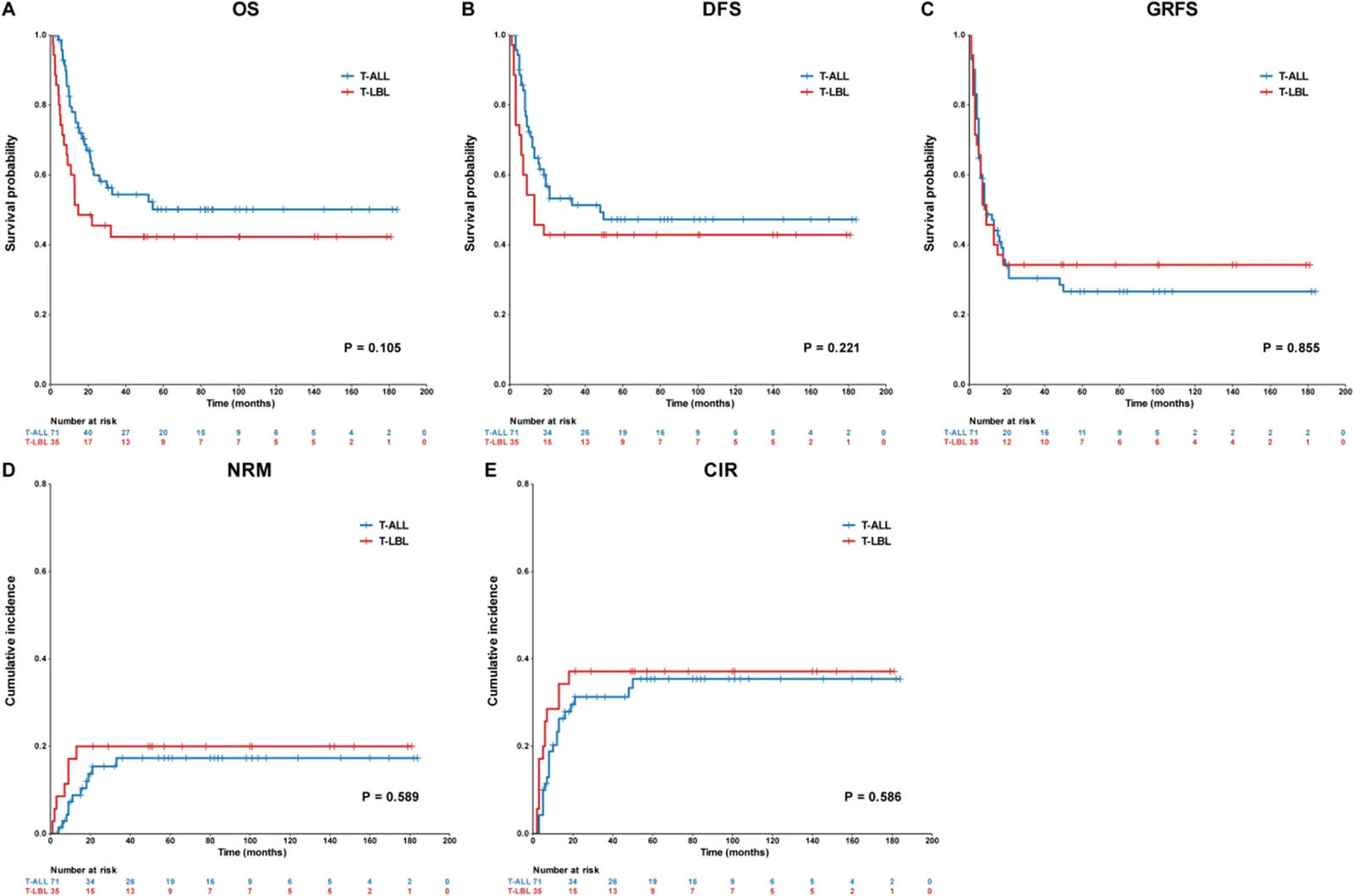
Clinical outcomes of T-ALL and T-LBL patients after allo-HSCT. (**A**) OS, (**B**) DFS, (**C**) GRFS, (**D**) cumulative incidence of NRM, (**E**) CIR in patients with T-ALL or T-LBL. T-ALL, T-cell acute lymphoblastic leukemia; T-LBL, T-cell lymphoblastic lymphoma; allo-HSCT, allogeneic hematopoietic stem-cell transplantation; OS, overall survival; DFS, disease-free survival; GRFS, graft-versus-host disease–free, relapse–free survival; NRM, non-relapse mortality; CIR, cumulative incidence of relapse

The clinical outcomes after allo-HSCT for all patients are summarized in Table 2. At 3 years after allo-HSCT, OS was 54.4% (95% CI, 41.2–65.9%) in the T-ALL group and 42.3% (95% CI, 25.8–57.9%) in the T-LBL group (*P* = 0.105; Fig. 2A), while DFS was 51.4% (95% CI, 38.5–62.8%) and 42.9% (95% CI, 26.4–58.3%), respectively (*P* = 0.221; Fig. 2B). At 3 years, GRFS was 30.5% (95% CI, 21.0–44.2%) in the T-ALL group and 34.3% (95% CI, 21.7–54.2%) in the T-LBL group, with no significant difference between the two groups (*P* = 0.855; Fig. 2C). The 3-year NRM was 17.3% (95% CI, 9.9–29.3%) and 20.0% (95% CI, 10.0–37.8%), respectively (*P* = 0.589; Fig. 2D). The 3-year CIR was 31.3% (95% CI, 21.5–44.1%) in patients with T-ALL and 37.1% (95% CI, 23.4–55.5%) in patients with T-LBL (*P* = 0.586; Fig. 2E).

**Table 2:** Clinical outcomes of T-ALL and T-LBL patients after allo-HSCT

Outcome	Time point	T-ALL, % (95% CI)	T-LBL, % (95% CI)	*P* value
		(*n* = 71)	(*n* = 35)	
Overall Survival	1-year	78.0% (66.1–86.1)	60.0% (42.0–74.0)	0.105
	2-year	59.9% (46.8–70.8)	45.5% (28.7–60.9)	
	3-year	54.4% (41.2–65.9)	42.3% (25.8–57.9)	
	5-year	50.1% (36.8–62.1)	42.3% (25.8–57.9)	
Disease-Free Survival	1-year	67.8% (55.3–77.5)	54.3% (36.6–69.0)	0.221
	2-year	53.3% (40.5–64.5)	42.9% (26.4–58.3)	
	3-year	51.4% (38.5–62.8)	42.9% (26.4–58.3)	
	5-year	47.3% (34.4–59.1)	42.9% (26.4–58.3)	
Non-relapse mortality	1-year	8.8% (4.0–18.7)	17.1% (8.0–34.6)	0.589
	2-year	15.4% (8.5–26.9)	20.0% (10.0–37.8)	
	3-year	17.3% (9.9–29.3)	20.0% (10.0–37.8)	
	5-year	17.3% (9.9–29.3)	20.0% (10.0–37.8)	
GVHD-free, relapse-free survival	1-year	47.2% (36.8–60.5)	45.7% (31.9–65.6)	0.855
	2-year	30.5% (21.0-44.2)	34.3% (21.7–54.2)	
	3-year	30.5% (21.0-44.2)	34.3% (21.7–54.2)	
	5-year	26.7% (17.6–40.4)	34.3% (21.7–54.2)	
Relapse	1-year	23.3% (14.9–35.4)	28.6% (16.4–46.9)	0.586
	2-year	31.3% (21.5–44.1)	37.1% (23.4–55.5)	
	3-year	31.3% (21.5–44.1)	37.1% (23.4–55.5)	
	5-year	35.4% (24.9–48.8)	37.1% (23.4–55.5)	
Grade II-IV aGVHD	30 days	19.7% (10.4–29.0)	20.0% (6.5–33.5)	0.274
	60 days	31.0% (20.1–41.8)	28.6% (13.3–43.8)	
	100 days	40.8% (29.3–52.4)	28.6% (13.3–43.8)	
Grade III-IV aGVHD	30 days	5.6% (0.2–11.0)	0.0% (0.0–0.0)	0.270
	60 days	8.5% (1.9–15.0)	5.7% (0.0–13.5)	
	100 days	12.7% (4.9–20.5)	5.7% (0.0–13.5)	
Any NIH cGVHD	1-year	23.0% (13.1–33.0)	5.7% (0.0-13.6)	0.033
	2-year	26.5% (15.8–37.1)	8.6% (0.0-18.1)	
	3-year	26.5% (15.8–37.1)	8.6% (0.0-18.1)	
	5-year	26.5% (15.8–37.1)	8.6% (0.0-18.1)	
Moderate-severe cGVHD	1-year	14.5% (6.1–22.8)	2.9% (0.0-8.5)	0.051
	2-year	16.1% (7.3–25.0)	2.9% (0.0-8.5)	
	3-year	16.1% (7.3–25.0)	2.9% (0.0-8.5)	
	5-year	16.1% (7.3–25.0)	2.9% (0.0-8.5)	

*T-ALL* T-cell acute lymphoblastic leukemia, *T-LBL *T-cell lymphoblastic lymphoma, *allo-HSCT *allogeneic hematopoietic stem-cell transplantation, *CI *confidence interval, *GVHD *graft-versus-host disease, *aGVHD *acute graft-versus-host disease, *cGVHD *chronic graft-versus-host disease, *NIH *National Institutes of Health

### GVHD outcomes in the overall cohort

**Fig. 3:**
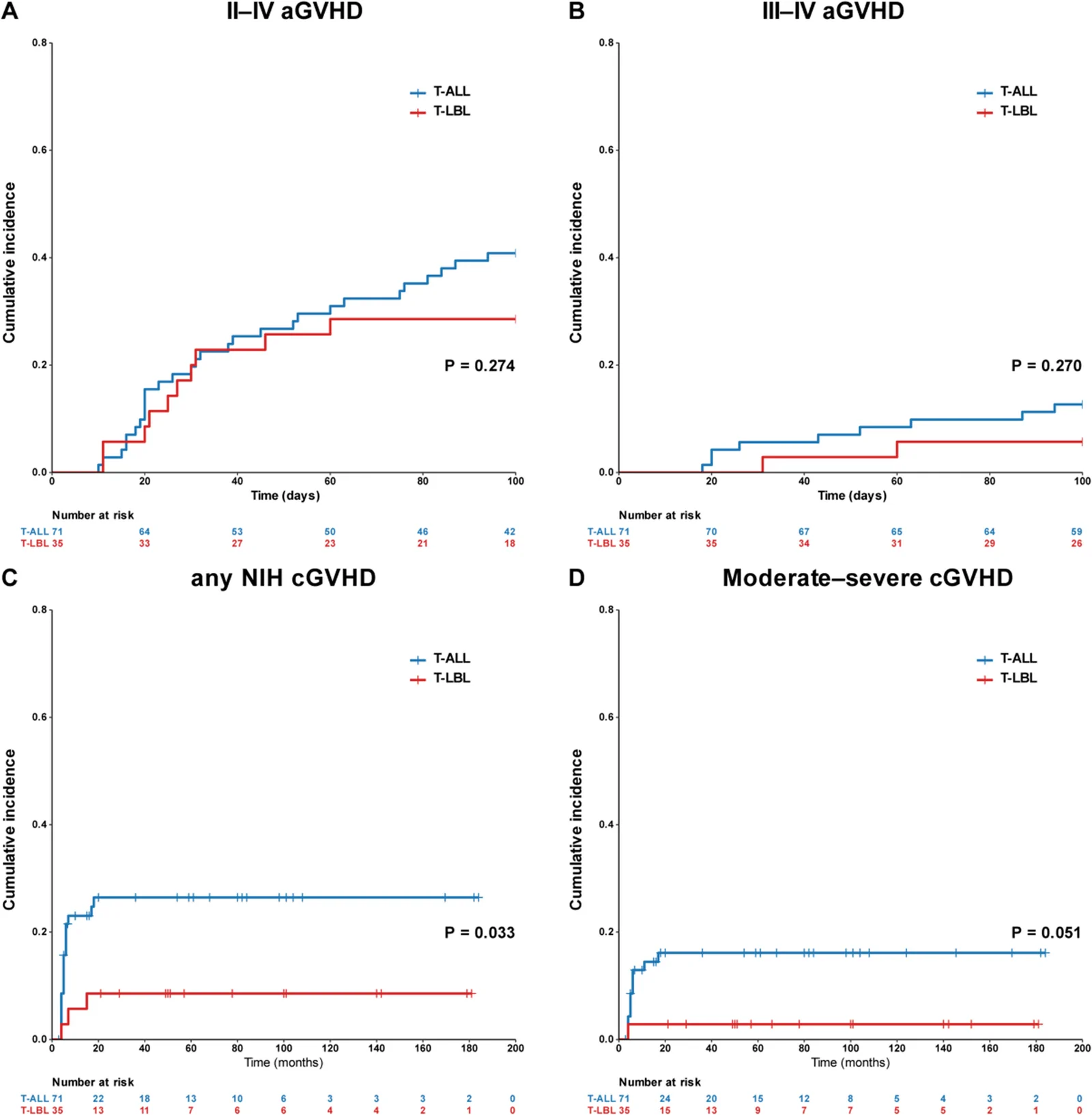
Cumulative incidence of acute and chronic graft-versus-host disease in patients with T-ALL and T-LBL after allo-HSCT. **A** The 100-day cumulative incidence of grade II–IV aGVHD. **B** The 100-day cumulative incidence of grade III–IV aGVHD. **C** The cumulative incidence of any NIH cGVHD. **D** The cumulative incidence of moderate to severe cGVHD, including progression from initially mild cGVHD

The cumulative incidence estimates for aGVHD and cGVHD are summarized in Table 2. The 100-day cumulative incidence of grade II–IV aGVHD was 40.8% (95% CI, 29.3–52.4%) in patients with T-ALL and 28.6% (95% CI, 13.3–43.8%) in patients with T-LBL (*P* = 0.274; Fig. 3A). Similarly, the 100-day cumulative incidence of grade III–IV aGVHD was 12.7% (95% CI, 4.9–20.5%) in the T-ALL group and 5.7% (95% CI, 0.0–13.5%) in the T-LBL group, with no statistically significant difference between the two groups (*P* = 0.270; Fig. 3B). At 3 years after allo-HSCT, the cumulative incidence of any NIH cGVHD was 26.5% (95% CI, 15.8–37.1%) in patients with T-ALL and 8.6% (95% CI, 0.0–18.1%) in those with T-LBL (*P* = 0.033; Fig. 3C). The 3-year cumulative incidence of moderate to severe cGVHD was 16.1% (95% CI, 7.3–25.0%) in the T-ALL group and 2.9% (95% CI, 0.0–8.5%) in the T-LBL group (*P* = 0.051; Fig. 3D). The distribution of NIH cGVHD severity at first diagnosis is summarized in Table S4.

### Propensity score matching and sensitivity analyses

As several baseline characteristics differed between the two groups, propensity score matching was used as a sensitivity analysis. The 2:1 PSM cohort included 70 patients, comprising 44 with T-ALL and 26 with T-LBL. All eight covariates included in the propensity score model had an absolute standardized mean difference below 0.10 after matching (Table S5; Figure S1). The strict 1:1 PSM analysis yielded 52 patients, with 26 in each group. In this cohort, hematopoietic cell transplantation comorbidity index (HCT-CI) had an absolute standardized mean difference (|SMD|) of 0.234, whereas the other covariates in the propensity score model had values below 0.10 (Table S5; Figure S2). The standardized mean differences (SMDs) of all reported baseline variables and the baseline characteristics of both matched cohorts are presented in Tables S5 and S6, respectively. Clinical outcomes in both PSM cohorts are summarized in the Table S7. OS, DFS, CIR, NRM, GRFS, and acute GVHD outcomes did not differ significantly in either PSM cohort. Besides, moderate to severe cGVHD was less frequent in T-LBL in the 2:1 PSM cohort (*P* = 0.031) and the strict 1:1 PSM cohort (*P* = 0.038). The cumulative incidence of any NIH cGVHD did not differ significantly between T-ALL and T-LBL in either PSM cohort.

### Prognostic factors

The results of univariate and multivariate analyses are summarized in Tables S8–S9 and Table 3. Diagnosis was not associated with OS, DFS, CIR, NRM, GRFS, grade II–IV aGVHD, or any NIH cGVHD in the multivariable analyses. Female sex was associated with better OS (HR, 0.39; 95% CI, 0.19–0.83; *P* = 0.014) and DFS (HR, 0.47; 95% CI, 0.23–0.93; *P* = 0.031). A higher HCT-CI was associated with worse OS (HR, 1.85; 95% CI, 1.10–3.11; *P* = 0.021), DFS (HR, 1.85; 95% CI, 1.11–3.06; *P* = 0.017), and GRFS (HR, 2.20; 95% CI, 1.43–3.40; *P* < 0.001). Transplantation during 2019–2025 was associated with better GRFS (HR, 0.54; 95% CI, 0.31–0.95; *P* = 0.033). HLA-haploidentical related donation was associated with a lower incidence of any NIH cGVHD than HLA-matched sibling donation (sHR, 0.28; 95% CI, 0.11–0.68; *P* = 0.005).

**Table 3:** Multivariable regression models for clinical outcomes in all patients

Outcome	Variable	HR or sHR (95% CI)	*P* value
OS	Diagnosis, T-LBL (ref. T-ALL)	1.35 (0.74–2.46)	0.321
	Age at allo-HSCT, per 10 years	1.14 (0.85–1.52)	0.390
	Sex, female (ref. male)	0.39 (0.19–0.83)	0.014
	Disease status, non-CR (ref. CR)	1.13 (0.46–2.82)	0.787
	HCT-CI, per category increase (0, 1, or ≥ 2)	1.85 (1.10–3.11)	0.021
	Transplant era, 2019–2025 (ref. 2010–2018)	0.55 (0.28–1.06)	0.073
DFS	Diagnosis, T-LBL (ref. T-ALL)	1.26 (0.70–2.26)	0.446
	Age at allo-HSCT, per 10 years	1.14 (0.87–1.49)	0.349
	Sex, female (ref. male)	0.47 (0.23–0.93)	0.031
	Disease status, non-CR (ref. CR)	1.08 (0.44–2.66)	0.870
	HCT-CI, per category increase (0, 1, or ≥ 2)	1.85 (1.11–3.06)	0.017
	Transplant era, 2019–2025 (ref. 2010–2018)	0.62 (0.33–1.17)	0.143
CIR	Diagnosis, T-LBL (ref. T-ALL)	0.98 (0.49–1.99)	0.960
	Age at allo-HSCT, per 10 years	0.99 (0.72–1.35)	0.944
	Disease status, non-CR (ref. CR)	2.08 (0.89–4.85)	0.089
	HCT-CI, per category increase (0, 1, or ≥ 2)	1.72 (0.99–2.99)	0.056
	Transplant era, 2019–2025 (ref. 2010–2018)	1.29 (0.66–2.53)	0.452
NRM	Diagnosis, T-LBL (ref. T-ALL)	1.18 (0.48–2.90)	0.725
	HCT-CI, per category increase (0, 1, or ≥ 2)	1.16 (0.34–3.91)	0.810
	Transplant era, 2019–2025 (ref. 2010–2018)	0.45 (0.11–1.75)	0.247
GRFS	Diagnosis, T-LBL (ref. T-ALL)	0.75 (0.44–1.28)	0.285
	Age at allo-HSCT, per 10 years	1.09 (0.86–1.38)	0.486
	Sex, female (ref. male)	0.61 (0.34–1.07)	0.086
	Disease status, non-CR (ref. CR)	1.24 (0.57–2.69)	0.592
	HCT-CI, per category increase (0, 1, or ≥ 2)	2.20 (1.43–3.40)	< 0.001
	Transplant era, 2019–2025 (ref. 2010–2018)	0.54 (0.31–0.95)	0.033
Grade II–IV aGVHD	Diagnosis, T-LBL (ref. T-ALL)	0.73 (0.35–1.50)	0.391
	Age at allo-HSCT, per 10 years	0.95 (0.74–1.23)	0.722
	Transplant era, 2019–2025 (ref. 2010–2018)	1.76 (0.93–3.36)	0.084
Any NIH cGVHD	Diagnosis, T-LBL (ref. T-ALL)	0.34 (0.11–1.09)	0.070
	Donor type, HID (ref. MSD)	0.28 (0.11–0.68)	0.005
	Donor type, MUD (ref. MSD)	0.18 (0.03–1.21)	0.078

HRs are reported for OS, DFS, and GRFS. sHRs are reported for CIR, NRM, and GVHD. Grade III–IV aGVHD was not included in the multivariable analysis because only 11 events occurred

*HR *hazard ratio, *sHR *subdistribution hazard ratio, *CI *confidence interval, *OS *overall survival, *DFS *disease-free survival, *GRFS *graft-versus-host disease-free, relapse-free survival, *NRM *non-relapse mortality, *CIR *cumulative incidence of relapse, *aGVHD *acute graft-versus-host disease, *NIH *National Institutes of Health, *cGVHD *chronic graft-versus-host disease, *T-ALL *T-cell acute lymphoblastic leukemia, *T-LBL *T-cell lymphoblastic lymphoma, *allo-HSCT *allogeneic hematopoietic stem cell transplantation, *CR *complete remission, *non-CR *non-complete remission, *HCT-CI *hematopoietic cell transplantation comorbidity index, *MSD * HLA-matched sibling donor, *HID *HLA-haploidentical related donor, *MUD *HLA-matched unrelated donor

We further examined whether the effects of diagnosis changed during follow-up. Schoenfeld residual tests for the univariable and multivariable Cox models are presented in Tables S10 and S11, respectively. The corresponding tests among patients in CR with evaluable MRD are presented in Table S12. Multivariable Cox and Fine–Gray regression models incorporating an interaction between diagnosis and time showed that the HR or sHR for diagnosis varied over time for OS (interaction *P* = 0.011), DFS (interaction *P* = 0.009), CIR (interaction *P* = 0.032), and NRM (interaction *P* = 0.008; Table 4). Time specific HRs for covariates other than diagnosis that did not meet the proportional hazards assumption in univariable analyses are presented in Table S13. The complete multivariable Cox and Fine–Gray models incorporating an interaction between diagnosis and time are presented in Table S14. T-LBL was associated with worse OS at 6 months (hazard ratio at the specified time [HR(t)], 2.41; 95% CI, 1.10–5.28; *P* = 0.028). At 24 months, T-LBL was associated with a lower time specific sHR for NRM (subdistribution hazard ratio at the specified time [sHR(t)], 0.14; 95% CI, 0.02–0.98; *P* = 0.047). At 36 months, T-LBL was associated with a lower time specific HR for DFS (HR(t), 0.16; 95% CI, 0.03–0.91; *P* = 0.039) and a lower time specific sHR for NRM (subdistribution hazard ratio at the specified time [sHR(t)], 0.06; 95% CI, 0.01–0.72; *P* = 0.026). No other time specific estimates for diagnosis at 6, 12, 24, or 36 months reached statistical significance. In addition, among patients in CR, multivariable Cox models including MRD showed that the HR for diagnosis varied over time for OS and DFS (interaction *P* = 0.013 and *P* = 0.016, respectively; Table S15). T-LBL was associated with worse OS at 6 months (HR(t), 2.50; 95% CI, 1.07–5.85; *P* = 0.035), whereas the other time specific estimates for diagnosis did not reach statistical significance. For the GVHD outcomes, time interaction analyses showed no significant interaction between diagnosis and time for grade II–IV aGVHD or any NIH cGVHD (Table S16).

**Table 4:** Univariable and multivariable effects of diagnosis over time on transplantation outcomes

Outcome	Time point	Effect measure	Univariable HR(t) or sHR(t) (95% CI)	*P* value	Interaction *P* value	Multivariable HR(t) or sHR(t) (95% CI)	*P* value	Interaction *P* value
OS	6 months	HR(t)	3.06 (1.44–6.52)	0.004	0.006	2.41 (1.10–5.28)	0.028	0.011
	12 months		1.18 (0.60–2.33)	0.623		1.01 (0.50–2.03)	0.984	
	24 months		0.43 (0.14–1.37)	0.156		0.40 (0.13–1.27)	0.120	
	36 months		0.24 (0.05–1.09)	0.065		0.23 (0.05–1.06)	0.059	
DFS	6 months	HR(t)	1.82 (0.98–3.37)	0.058	0.005	1.58 (0.82–3.03)	0.170	0.009
	12 months		0.72 (0.32–1.63)	0.430		0.67 (0.29–1.55)	0.347	
	24 months		0.27 (0.07–1.07)	0.063		0.27 (0.07–1.08)	0.064	
	36 months		0.15 (0.03–0.87)	0.035		0.16 (0.03–0.91)	0.039	
CIR	6 months	sHR(t)	1.43 (0.71–2.87)	0.319	0.031	1.16 (0.56–2.41)	0.689	0.032
	12 months		0.62 (0.22–1.78)	0.378		0.52 (0.19–1.43)	0.203	
	24 months		0.26 (0.05–1.46)	0.126		0.22 (0.04–1.15)	0.073	
	36 months		0.15 (0.02–1.34)	0.091		0.13 (0.02–1.06)	0.057	
NRM	6 months	sHR(t)	2.33 (0.70–7.74)	0.168	0.007	2.06 (0.63–6.75)	0.234	0.008
	12 months		0.63 (0.18–2.23)	0.469		0.56 (0.16–1.96)	0.368	
	24 months		0.16 (0.02–1.08)	0.060		0.14 (0.02–0.98)	0.047	
	36 months		0.07 (0.01–0.79)	0.031		0.06 (0.01–0.72)	0.026	

All analyses compare T-LBL with T-ALL, with T-ALL as the reference group. The multivariable analyses included the covariates shown for each outcome in Table 3. The OS model included interactions of log(1 + time) with diagnosis and transplant era, whereas the DFS, CIR, and NRM models included an interaction with diagnosis only. The reported interaction P value indicates whether the association between diagnosis and the outcome changed during follow-up

* HR(t) *hazard ratio at the specified time, *sHR(t) *subdistribution hazard ratio at the specified time, *CI *confidence interval, *OS *overall survival, *DFS *disease-free survival, *CIR *cumulative incidence of relapse, *NRM *non-relapse mortality, *T-ALL *T-cell acute lymphoblastic leukemia, *T-LBL *T-cell lymphoblastic lymphoma

The complete case sensitivity analyses involving WBC and LDH are summarized in Table S17. The results showed that adding WBC, LDH, or both did not materially change the estimated association between diagnosis and OS, DFS, or GRFS. In the LDH complete case analysis for CIR, diagnosis was not significantly associated with CIR (sHR, 1.26; 95% CI, 0.59–2.68; *P* = 0.545), whereas elevated LDH was associated with a higher relapse incidence (sHR, 2.35; 95% CI, 1.09–5.09; *P* = 0.029). The multivariable analyses among patients in CR with evaluable MRD are summarized in Table S18. Among 94 patients in CR, diagnosis was not significantly associated with OS (HR, 1.37; 95% CI, 0.71–2.63; *P* = 0.344) or DFS (HR, 1.28; 95% CI, 0.67–2.44; *P* = 0.456) after adjustment for MRD and the other covariates included in the model. Similarly, MRD negativity was not significantly associated with OS (HR, 0.85; 95% CI, 0.40–1.82; *P* = 0.678) or DFS (HR, 0.80; 95% CI, 0.38–1.66; *P* = 0.548). The proportional hazards tests for these models are shown in Table S12, and the corresponding results over time are shown in Table S15.

### Restricted mean survival time and restricted mean time lost analyses

Restricted mean analyses are shown in Table 5. At 36 months, OS RMST was 26.28 months in T-ALL and 20.50 months in T-LBL, with a difference of − 5.79 months (95% CI, − 11.38 to − 0.19; *P* = 0.043). However, RMST for DFS and GRFS and RMTL for CIR, NRM, and acute GVHD did not differ significantly between the two groups. In contrast, RMTL was lower in T-LBL for any NIH cGVHD (7.77 months vs. 2.34 months; *P* = 0.008) and moderate to severe cGVHD (4.71 months vs. 0.91 months; *P* = 0.017). The RMTL findings for any NIH cGVHD and moderate to severe cGVHD were considered exploratory because the numbers of events for both outcomes were limited.

**Table 5:** Restricted mean survival time and restricted mean time lost according to diagnosis

Outcome	Measure	Truncation time	T-ALL (*n* = 71), restricted mean (95% CI)	T-LBL (*n* = 35), restricted mean (95% CI)	Difference, T-LBL minus T-ALL (95% CI)	*P* value
OS	RMST	36 months	26.28 (23.42 to 29.14)	20.50 (15.69 to 25.30)	−5.79 (− 11.38 to − 0.19)	0.043
DFS		36 months	23.93 (20.74 to 27.12)	19.09 (14.10 to 24.07)	−4.85 (− 10.77 to 1.08)	0.109
GRFS		36 months	16.39 (13.08 to 19.70)	16.37 (11.51 to 21.23)	−0.02 (− 5.90 to 5.86)	0.995
CIR	RMTL	36 months	8.36 (5.33 to 11.39)	10.97 (6.14 to 15.80)	2.61 (− 3.09 to 8.31)	0.370
NRM		36 months	3.70 (1.58 to 5.82)	5.94 (1.96 to 9.93)	2.24 (− 2.27 to 6.75)	0.330
Grade II to IV aGVHD		100 days	24.31 (16.50 to 32.12)	20.51 (9.47 to 31.56)	−3.80 (− 17.32 to 9.73)	0.582
Grade III to IV aGVHD		100 days	6.72 (2.02 to 11.41)	3.11 (0.00 to 7.46)	−3.60 (− 10.00 to 2.79)	0.270
Any NIH cGVHD		36 months	7.77 (4.66 to 10.88)	2.34 (0.00 to 4.92)	−5.43 (− 9.46 to − 1.39)	0.008
Moderate to severe cGVHD		36 months	4.71 (2.14 to 7.27)	0.91 (0.00 to 2.68)	−3.79 (− 6.91 to − 0.68)	0.017

Values are restricted means with 95% confidence intervals. Differences are T-LBL minus T-ALL. Negative values indicate shorter RMST or less RMTL in T-LBL. Chronic GVHD results are exploratory because of the small number of events

*aGVHD *acute graft-versus-host disease, *cGVHD *chronic graft-versus-host disease, *CI *confidence interval, *CIR *cumulative incidence of relapse, *DFS *disease-free survival, *GRFS *graft-versus-host disease-free, relapse-free survival, *NIH *National Institutes of Health, *NRM *non-relapse mortality, *OS *overall survival, *RMST *restricted mean survival time, *RMTL *restricted mean time lost, *T-ALL *T-cell acute lymphoblastic leukemia, *T-LBL *T-cell lymphoblastic lymphoma

### Subgroup analysis in patients transplanted in CR1

To further assess outcomes under a comparable disease status at transplantation, we performed a subgroup analysis among 62 T-ALL and 24 T-LBL patients who achieved CR1 before allo-HSCT (Table S7). The 3-year OS rates were 56.8% (95% CI, 42.9–68.5%) in patients with T-ALL and 49.7% (95% CI, 28.7–67.6%) in those with T-LBL (*P* = 0.454). Similarly, the 3-year DFS rates were 54.4% (95% CI, 40.8–66.2%) and 50.0% (95% CI, 29.1–67.8%) for T-ALL and T-LBL, respectively (*P* = 0.615). The 3-year cumulative incidence of relapse was 28.2% (95% CI, 18.5–41.6%) in T-ALL and 25.0% (95% CI, 11.9–48.1%) in T-LBL patients (*P* = 0.643). The corresponding 3-year GRFS rates were 34.7% (95% CI, 22.8–47.0%) for T-ALL and 37.5% (95% CI, 19.0–56.0%) for T-LBL (*P* = 0.664), while the 3-year NRM rates were 17.4% (95% CI, 9.6–30.2%) and 25.0% (95% CI, 11.9–48.1%), respectively (*P* = 0.335).

The 100-day cumulative incidences of grade II–IV aGVHD (41.9% vs. 33.3%; *P* = 0.552) and grade III–IV aGVHD (12.9% vs. 4.2%; *P* = 0.236) were comparable between patients with T-ALL and T-LBL (Table S7). The 3-year cumulative incidence of any NIH cGVHD was 26.6% in patients with T-ALL and 8.3% in those with T-LBL (*P* = 0.061), while the 3-year cumulative incidence of moderate to severe cGVHD was 16.6% in patients with T-ALL and 0% in those with T-LBL (*P* = 0.035; Table S7).

## Discussion

In the current study, we retrospectively analyzed and compared the survival and other transplant-related outcomes of patients with T-ALL and T-LBL who underwent allo-HSCT. Overall, OS, DFS, CIR, NRM, and GRFS after allo-HSCT did not differ significantly between patients with T-ALL and T-LBL. Furthermore, diagnosis was not significantly associated with OS, DFS, CIR, NRM, or GRFS in multivariable analyses. The PSM analyses and CR1 subgroup analysis also showed no significant differences in these outcomes between T-ALL and T-LBL. However, the association between diagnosis and OS, DFS, CIR, and NRM changed during follow-up. T-LBL was associated with worse early OS, whereas better DFS and lower NRM were observed later. These findings suggest that the overall prognosis after allo-HSCT did not differ significantly between T-ALL and T-LBL, but the risks may differ at different times after transplantation. Nevertheless, further multicenter studies with larger sample sizes are needed to confirm these findings. Despite the relatively small cohort size due to the rarity of these diseases, our study provides real-world evidence that contributes to the limited literature directly comparing post-transplant outcomes between T-ALL and T-LBL.

Published studies have reported varying outcomes after allo-HSCT in patients with T-ALL and T-LBL, with 3-year OS rates ranging from 35.0% to 55.7% among patients with T-ALL [22, 40], and from 41.0% to 56.2% among those with T-LBL [16, 22, 41]. The OS estimates observed in our cohort were concordant with previously reported results, supporting the external validity of our findings. Notably, most prior reports evaluated T-ALL or T-LBL separately rather than directly comparing outcomes between the two entities. In addition, differences in baseline risk profiles, disease status before transplantation, conditioning regimens, and donor types across studies further complicate interpretation [14, 40, 42–44]. Therefore, the present study was designed to perform a direct head-to-head comparison of clinical outcomes after allo-HSCT between patients with T-ALL and T-LBL using complementary statistical methods.

In the overall cohort, OS, DFS, CIR, NRM, and GRFS did not differ significantly between T-ALL and T-LBL. Diagnosis was also not associated with these outcomes in the multivariable analyses. As disease and transplant characteristics differed between the groups, PSM was used to reduce the potential influence of these differences. Furthermore, OS, DFS, CIR, NRM, and GRFS did not differ significantly between T-ALL and T-LBL in both PSM cohorts or in the CR1 subgroup. Notably, moderate to severe cGVHD was less frequent in T-LBL in both PSM cohorts and the CR1 subgroup. However, no moderate to severe cGVHD event occurred in T-LBL and the total number of events was small, so this finding should be interpreted cautiously. Any NIH cGVHD was also less frequent in T-LBL in the overall cohort. This association was not significant after donor type was included in the multivariable model, suggesting that transplant related factors may have contributed to the difference. The complete case analyses involving WBC and LDH did not materially change the associations between diagnosis and OS, DFS, or GRFS, and diagnosis was not significantly associated with CIR in patients with recorded LDH. The OS and DFS results were also similar in patients in CR with evaluable MRD.

We next assessed whether the associations between diagnosis and the clinical outcomes remained constant during follow-up. Specifically, Schoenfeld residual tests for OS and DFS and time interaction tests in the Fine–Gray models for CIR and NRM indicated that the effects of diagnosis changed during follow-up. These associations were therefore examined at selected time points. The results showed that T-LBL was associated with worse OS at 6 months, whereas a lower time specific sHR for NRM was observed at 24 and 36 months and a lower time specific HR for DFS was observed at 36 months. This pattern suggests an early survival disadvantage for T-LBL, which was also reflected by the lower OS RMST over the first 36 months, whereas the later relative risk estimates for DFS and NRM favored T-LBL. Of note, the wide confidence intervals for the 36-month DFS and NRM estimates indicated limited precision. Moreover, T-LBL also showed lower 36-month RMTL estimates for any NIH cGVHD and moderate to severe cGVHD. However, the RMTL results were exploratory because the numbers of these events were limited. Overall, comparisons of OS, DFS, CIR, NRM, and GRFS did not show statistically significant differences between the two diseases, but time dependent analyses indicated that the effects of diagnosis on several outcomes changed during follow-up.

In the multivariable analyses, a higher HCT-CI category was associated with worse OS and DFS, supporting the clinical value of comorbidity assessment before transplantation and helping identify patients who may require closer monitoring and supportive care. Although statistical significance was not reached, the magnitude of the association between non-CR status and CIR suggests that disease control at transplantation may remain clinically relevant. Accordingly, achieving deep remission before allo-HSCT remains an important clinical goal. In this context, nelarabine [45–47], daratumumab [48–50], and CD7 chimeric antigen receptor (CAR)-T cell therapy, with or without bridging transplantation [51, 52], have shown encouraging results in relapsed or refractory T-cell malignancies and may provide additional treatment options. For NRM, none of the variables included in the multivariable model was significantly associated with the outcome, possibly because the limited number of events reduced the precision of the estimates. As a composite endpoint integrating relapse, death, and major transplant-related complications, GRFS reflects both disease burden and factors related to transplantation. In our cohort, a higher HCT-CI category was also associated with worse GRFS, while the better GRFS observed during 2019–2025 may reflect broader advances in transplant care, highlighting the relevance of both comorbidity burden and evolving transplant practices to this composite outcome.

Existing studies have indicated that diverse genetic abnormalities can impact the treatment response of patients with T-ALL or T-LBL [11, 53–55]. Meanwhile, other studies have also shown that the prognostic significance of certain genes differs between T-ALL and T-LBL patients [6, 56]. Exploring the genetic variations and underlying mechanisms of these diseases offers potential for developing tailored therapies, thereby highlighting the importance of genetic profiling in their management. In our study, we summarized the genetic abnormalities identified in bone marrow samples from patients within our cohort. However, it is important to acknowledge that our center’s historical data on these specific genetic tests are incomplete, as genetic abnormalities were not a primary focus in the past [2, 54, 57], and we were only able to obtain genetic abnormality results from bone marrow samples of a limited number of T-LBL patients. Our results indicated that NOTCH1 mutation was a common genetic abnormality in T-ALL, in line with the high frequency previously reported [58]. Mutation in NOTCH1 has been shown to trigger active NOTCH1 signaling, which plays a crucial role in the pathogenesis of T-ALL [2]. Furthermore, NOTCH1 mutations were reported to be associated with a favorable outcome and improved steroid responsiveness, highlighting the significance of NOTCH1 in treatment response and prognosis in T-ALL [54, 58–60]. We also identified PHF6 mutation as a prevalent genetic aberration among patients with T-ALL. Although less frequent, this alteration has also been identified in patients with T-LBL. Interestingly, prior studies have reported discordant biological and clinical effects of this alteration between T-ALL and T-LBL. While PHF6 mutations are associated with reduced prednisolone sensitivity in T-ALL cell lines, they do not appear to adversely affect DFS or OS in adult T-ALL patients [5, 6], and have even been reported to be associated with favorable outcomes in adult T-LBL [7]. We also identified genetic abnormalities such as RAD21 and ASXL3 mutations in patients with T-ALL or T-LBL, which have not been specifically reported in these disease entities. Notably, these alterations have been implicated in the pathogenesis and prognosis of other hematological malignancies [61, 62]. However, due to the limited number of patients, the treatment response and prognostic impact associated with different genetic abnormalities could not be systematically evaluated in our study.

Our study has several limitations. Its retrospective, single-center design and modest sample size may limit generalizability and permit residual confounding. The propensity score model was restricted to clinically relevant covariates appropriate for the available sample size. Although the 2:1 PSM analysis balanced all variables included in the model and the strict 1:1 analysis yielded unchanged main survival and relapse conclusions, residual imbalances remained, particularly for HCT-CI in the strict 1:1 cohort. Thus, PSM reduced measured confounding but could not eliminate all baseline differences or unmeasured confounding. Besides, data on genetic aberrations were not routinely collected during the study period, particularly for T-LBL patients. In addition, the median age was 25 years in the present study, suggesting that most patients were adolescents and young adults, and the safety and efficacy of allo-HSCT in older patients should be further explored. Furthermore, the small number of chronic GVHD events limited the interpretation of these results, and the wide confidence intervals for the 36-month time specific estimates of DFS and NRM indicated limited precision. Finally, this study was not designed to establish formal equivalence between T-ALL and T-LBL. Nevertheless, this direct comparison provides clinically informative estimates of outcome differences between T-ALL and T-LBL in a rare transplant population.

## Conclusion

In summary, overall comparisons of OS, DFS, CIR, NRM, and GRFS showed no statistically significant differences between patients with T-ALL and T-LBL, although the effects of diagnosis on OS, DFS, CIR, and NRM varied during follow-up. These findings suggest that neither diagnosis had a consistent prognostic advantage, but the timing of posttransplant risk may differ. The results should be interpreted cautiously and confirmed in larger multicenter studies. Diagnostic subtype alone should not be used to predict posttransplant prognosis.

## Supplementary Information

Below is the link to the electronic supplementary material.

Supplementary Material 1

Supplementary Material 2

Supplementary Material 3

## Data Availability

The study-related data will be made available upon request.
