# Supplementary material for: Clinical outcomes of T-cell acute lymphoblastic leukemia versus T-cell lymphoblastic lymphoma after allogeneic hematopoietic stem cell transplantation
Source: Ann Hematol. 2026 Sep 29;105(12):503. doi: 10.1007/s00277-026-07295-4 (PMC13743562; doi:10.1007/s00277-026-07295-4)
Supplement: Supplementary file 1 [file 277_2026_7295_MOESM1_ESM.docx]

**Supplementary Figures**

**Supplementary Figure 1. Covariate balance before and after the 2:1 propensity score matching analysis.**

The Love plot shows the absolute standardized mean differences (SMDs) for the covariates included in the propensity score model before and after matching. In the 2:1 PSM analysis, either one or two patients with T-ALL were matched to each patient with T-LBL. The 2:1 PSM cohort included 44 patients with T-ALL and 26 with T-LBL. The dashed line indicates an absolute SMD of 0.10. All displayed covariates had absolute SMDs below 0.10 after matching.

Abbreviations: allo-HSCT, allogeneic hematopoietic stem cell transplantation; CR, complete remission; HCT-CI, Hematopoietic Cell Transplantation Comorbidity Index; HID, HLA-haploidentical related donor; HLA, human leukocyte antigen; MSD, HLA-matched sibling donor; MUD, HLA-matched unrelated donor; PSM, propensity score matching; SMD, standardized mean difference; T-ALL, T-cell acute lymphoblastic leukemia; T-LBL, T-cell lymphoblastic lymphoma.

**Supplementary Figure 2. Covariate balance before and after strict 1:1 propensity score matching.**

The Love plot shows the absolute standardized mean differences (SMDs) for the covariates included in the propensity score model before and after strict 1:1 PSM. The strict 1:1 PSM cohort included 26 patients in each group. The dashed line indicates an absolute SMD of 0.10. HCT-CI remained imbalanced after matching (absolute SMD, 0.234), whereas all other displayed covariates had absolute SMDs below 0.10.

Abbreviations: allo-HSCT, allogeneic hematopoietic stem cell transplantation; CR, complete remission; HCT-CI, Hematopoietic Cell Transplantation Comorbidity Index; HID, HLA-haploidentical related donor; HLA, human leukocyte antigen; MSD, HLA-matched sibling donor; MUD, HLA-matched unrelated donor; PSM, propensity score matching; SMD, standardized mean difference; T-ALL, T-cell acute lymphoblastic leukemia; T-LBL, T-cell lymphoblastic lymphoma.
