# Supplementary material for: Clinical outcomes of T-cell acute lymphoblastic leukemia versus T-cell lymphoblastic lymphoma after allogeneic hematopoietic stem cell transplantation
Source: Ann Hematol. 2026 Sep 29;105(12):503. doi: 10.1007/s00277-026-07295-4 (PMC13743562; doi:10.1007/s00277-026-07295-4)
Supplement: Supplementary file 2 [file 277_2026_7295_MOESM2_ESM.docx]

**Supplementary Methods**

**Intrathecal therapy**

Intrathecal therapy was administered before or after allo-HSCT according to clinical indication and was classified as prophylactic or therapeutic. Therapeutic administrations were those given for documented central nervous system involvement. Receipt of intrathecal therapy was recorded only when at least one administration was explicitly documented. Regimens and the number of administrations were analyzed only among patients who received intrathecal therapy and had complete records of these administrations. Triple intrathecal therapy comprised methotrexate, cytarabine, and a glucocorticoid administered during the same procedure. Other regimens comprised cytarabine plus dexamethasone or methotrexate plus dexamethasone. Regimen categories were not mutually exclusive because individual patients could receive different regimens on separate occasions.

Intrathecal regimens were classified according to the agents administered during the same procedure. Among administrations with documented dose information, cytarabine was administered at 50 mg and dexamethasone at 5 mg. Methotrexate was administered at 10 or 15 mg. Dose information from outside hospital records was extracted when available. Missing doses were not inferred.

**Missing data and analytical samples**

Missing values for white blood cell count (WBC), lactic dehydrogenase (LDH), mediastinal involvement, and early T-cell precursor (ETP) immunophenotype were not imputed. Unknown ETP immunophenotype was treated as missing. Baseline comparisons, descriptive standardized mean difference calculations, and univariable regression analyses involving these variables included only patients with recorded values for the variable concerned. To limit overfitting, the propensity score model and primary multivariable outcome models were restricted to clinically relevant covariates appropriate for the available sample size and did not include WBC, LDH, mediastinal involvement, or ETP immunophenotype. Therefore, missing values for these variables did not reduce the samples used in these models. Separate complete case sensitivity analyses involving WBC and LDH are described below. MRD was not included in the primary multivariable models, and separate MRD analyses were restricted to patients in complete remission with evaluable MRD results. Two patients with T-ALL and no patients with T-LBL were lost to follow-up. They remained included in the analyses and were censored at their last documented follow-up for outcomes not observed before that date.

**Propensity score matching and first complete remission subgroup analysis**

Propensity scores were estimated by logistic regression using age at allo-HSCT, sex, disease status, HCT-CI grouped as 0, 1, and ≥2, donor recipient gender matching, time from diagnosis to allo-HSCT in months after natural log transformation, donor type, and transplant era. In the primary 2:1 PSM analysis, nearest neighbor matching without replacement matched either one or two patients with T-ALL to each patient with T-LBL. The caliper was 0.5 standard deviations of the logit of the propensity score. The strict 1:1 PSM analysis was used as a sensitivity analysis. Covariate balance was assessed using absolute standardized mean differences, with a value below 0.10 indicating adequate balance. Signed standardized mean differences were calculated as T-LBL minus T-ALL, and the Love plots display the corresponding absolute values. Outcomes in both PSM cohorts and the first complete remission subgroup were estimated using Kaplan–Meier or cumulative incidence methods and were compared using log rank or Gray tests. The first complete remission subgroup comprised patients from the original unmatched cohort.

**Univariable and multivariable regression analyses**

Univariable Cox regression was used to examine candidate clinical variables for OS, DFS, and GRFS. Univariable Fine–Gray regression was used for CIR, NRM, grade II–IV aGVHD, grade III–IV aGVHD, and any NIH cGVHD. T-ALL was the reference group in all analyses of diagnosis. The diagnosis coefficient therefore represents T-LBL compared with T-ALL. Continuous variables were analyzed using the units shown in the tables, and categorical variables used the stated reference categories.

During the revision, variable coding was reviewed to improve consistency and clinical interpretation. Age was analyzed as ≤35 years versus >35 years in the univariable analyses to remain consistent with the original analysis. It was analyzed as a continuous variable in the multivariable models to preserve information. The HR was reported per increase of 10 years for easier interpretation. Bone marrow blast percentage remained continuous as in the original analysis. Its HR was reported per increase of 10 percentage points. HCT-CI was entered as an ordered variable using categories of 0, 1, and ≥2 to preserve its clinical order and limit the number of parameters. Disease status was grouped as CR or non-CR because few patients and events were present in some categories. The same coding was used in the propensity score analysis where applicable. Model assumption assessments and time interaction analyses followed the coding of the corresponding regression models.

Covariates in the multivariable models were selected by considering the clinical question, clinical relevance, the number of observed events, and the univariable results rather than univariable P values alone. The Cox models for OS, DFS, and GRFS included diagnosis, age at allo-HSCT per 10 years, sex, disease status, HCT-CI entered as ordered categories of 0, 1, and ≥2, and transplant era. The Fine–Gray model for CIR included diagnosis, age, disease status, HCT-CI, and transplant era. The NRM model included diagnosis, HCT-CI, and transplant era. The grade II–IV aGVHD model included diagnosis, age, and transplant era. No multivariable model was used for grade III–IV aGVHD because only 11 events occurred.

The multivariable model for any NIH cGVHD included diagnosis and donor type, because the number of events did not support simultaneous adjustment for additional variables. Moderate to severe cGVHD was also evaluated as a separate endpoint. Mild cGVHD was not analyzed as a separate outcome because cGVHD severity could change over time. Any NIH cGVHD captured the initial occurrence of cGVHD. Moderate to severe cGVHD represented more clinically significant disease and included progression from mild cGVHD. Multivariable regression was not performed for moderate to severe cGVHD because the number of events was limited.

Mediastinal involvement and ETP immunophenotype were evaluated in their respective univariable analyses. They were not included in the primary multivariable models because they describe disease phenotype closely related to diagnosis and their inclusion would have increased model complexity.

**Sensitivity analyses for WBC and LDH**

Complete case sensitivity analyses assessed whether adding WBC or LDH changed the association between diagnosis and the outcomes. For OS, DFS, and GRFS, the corresponding primary Cox model was repeated among patients with recorded WBC, recorded LDH, or both. Within each sample, the model was first fitted without WBC or LDH and was then fitted after adding WBC, LDH, or both. The compared models were fitted in the same complete case cohort to avoid differences caused by changes in sample composition. WBC was categorized as >100 × 10⁹/L or ≤100 × 10⁹/L. LDH was categorized as elevated or normal.

For CIR, the primary Fine–Gray model was repeated among patients with recorded LDH. It was first fitted without LDH and then with LDH. A corresponding WBC analysis was not performed because only two WBC values were missing and WBC >100 × 10⁹/L occurred only in T-ALL. This provided insufficient overlap for stable estimation after adjustment for diagnosis. Additional WBC or LDH models were not performed for NRM or the GVHD outcomes because the limited numbers of events increased the risk of overfitting.

**MRD subgroup analyses**

Pretransplant MRD was evaluated among patients who were in complete remission and had an evaluable MRD result before allo-HSCT. Univariable Cox models assessed the association of MRD with OS, DFS, and GRFS. Given the exploratory nature of the MRD subgroup analysis and the smaller number of evaluable patients, multivariable analyses were limited to OS and DFS as the main survival outcomes. GRFS was assessed only in the univariable analysis.

The multivariable models included the same covariates as the corresponding primary models, except that disease status was omitted because all patients were in complete remission and MRD status was added. The models therefore included diagnosis, MRD, age at allo-HSCT per 10 years, sex, HCT-CI entered as ordered categories of 0, 1, and ≥2, and transplant era. Missing MRD values were not imputed.

**Assessment of proportional hazards and changes in effects over time**

For OS, DFS, and GRFS, the proportional hazards assumption was assessed using scaled Schoenfeld residuals. Each estimable univariable Cox model was assessed separately. In the primary multivariable models and the MRD subgroup models, the assumption was assessed for each covariate and for the model as a whole using the global test. A P value below 0.05 indicated that the proportional hazards assumption was not met.

A covariate that did not meet the proportional hazards assumption in a univariable model was evaluated in an additional model containing the covariate and its interaction with log(1+time). HRs with 95% CIs were estimated at 6, 12, 24, and 36 months. When the assumption was not met for a covariate in a multivariable model, the corresponding interaction was added while all other covariates were retained. When more than one covariate did not meet the assumption, their interactions were included together. Models without time interactions provided one adjusted HR for each covariate, whereas models with time interactions described changes in HRs during follow-up. No multivariable time interaction model was used for GRFS because neither the covariate specific tests nor the global test indicated that the proportional hazards assumption was not met.

Schoenfeld residual tests do not apply to Fine–Gray models. Changes in effects over time were therefore assessed using interactions with log(1+time). The diagnosis interaction was assessed in the univariable and multivariable models for CIR and NRM. For GVHD outcomes, time interactions were assessed only in the primary multivariable models to provide adjusted estimates while limiting model complexity. Diagnosis, age, and transplant era were assessed separately in the grade II–IV aGVHD model. Diagnosis and donor type were assessed separately in the any NIH cGVHD model. All covariates in the corresponding primary model were retained when each interaction was assessed. Time interaction models were not used for grade III–IV aGVHD or moderate to severe cGVHD because the numbers of events were limited. When an interaction indicated that an effect changed over time, sHRs with 95% CIs were estimated at clinically relevant time points.

**Restricted mean analyses**

RMST over the first 36 months was estimated for OS, DFS, and GRFS as the area under the Kaplan–Meier curve. RMTL was estimated as the area under the cumulative incidence curve. RMTL was evaluated over the first 36 months for CIR, NRM, any NIH cGVHD, and moderate to severe cGVHD and over the first 100 days for grade II–IV and grade III–IV aGVHD.

Differences were calculated as T-LBL minus T-ALL. A negative RMST difference indicates less mean survival or event free time in T-LBL. A positive RMTL difference indicates more mean time lost because of the corresponding event in T-LBL. Confidence intervals were calculated using standard variance estimates.
