# Supplementary material for: Clinical outcomes of T-cell acute lymphoblastic leukemia versus T-cell lymphoblastic lymphoma after allogeneic hematopoietic stem cell transplantation
Source: Ann Hematol. 2026 Sep 29;105(12):503. doi: 10.1007/s00277-026-07295-4 (PMC13743562; doi:10.1007/s00277-026-07295-4)
Supplement: Supplementary file 3 [file 277_2026_7295_MOESM3_ESM.docx]

**Supplementary Tables**

**Table S1. Missing data and analytical handling.**

| **Variable** | **Overall missing, n (%)** | **T-ALL missing, n (%)** | **T-LBL missing, n (%)** |
| --- | --- | --- | --- |
| WBC at diagnosis | 2 (1.9%) | 1 (1.4%) | 1 (2.9%) |
| LDH at diagnosis | 14 (13.2%) | 8 (11.3%) | 6 (17.1%) |
| Mediastinal involvement at diagnosis | 4 (3.8%) | 4 (5.6%) | 0 (0.0%) |
| ETP immunophenotype at diagnosis | 10 (9.4%) | 7 (9.9%) | 3 (8.6%) |

Missing values were not imputed. Analyses of individual variables included only patients with recorded values. Complete case analyses for WBC and LDH and analyses involving MRD are described in the Supplementary Methods.

Abbreviations: T-ALL, T-cell acute lymphoblastic leukemia; T-LBL, T-cell lymphoblastic lymphoma; WBC, white blood cell count; LDH, lactic dehydrogenase; ETP, early T-cell precursor; MRD, measurable residual disease.

**Table S2. Documentation and receipt of prophylactic and therapeutic intrathecal therapy before and after allo-HSCT.**

| **Characteristic** | **Overall (N=106)** | **T-ALL (n=71)** | **T-LBL (n=35)** | **Assessable, n** | **Not assessable, n** |
| --- | --- | --- | --- | --- | --- |
| Completeness of intrathecal therapy records, n (%) |  |  |  |  |  |
| Complete records | 75 (70.8%) | 48 (67.6%) | 27 (77.1%) | — | — |
| Incomplete outside-hospital records | 24 (22.6%) | 20 (28.2%) | 4 (11.4%) | — | — |
| Unable to ascertain from available records | 7 (6.6%) | 3 (4.2%) | 4 (11.4%) | — | — |
| Intrathecal therapy before allo-HSCT, n (%) |  |  |  |  |  |
| Prophylactic | 92 (92.9%) | 63 (92.6%) | 29 (93.5%) | Overall 99, T-ALL 68, T-LBL 31 | Overall 7, T-ALL 3, T-LBL 4 |
| Therapeutic | 4 (5.9%) | 3 (7.1%) | 1 (3.8%) | Overall 68, T-ALL 42, T-LBL 26 | Overall 38, T-ALL 29, T-LBL 9 |
| Intrathecal therapy after allo-HSCT, n (%) |  |  |  |  |  |
| Prophylactic | 10 (13.5%) | 5 (10.4%) | 5 (19.2%) | Overall 74, T-ALL 48, T-LBL 26 | Overall 32, T-ALL 23, T-LBL 9 |
| Therapeutic | 5 (6.8%) | 4 (8.5%) | 1 (3.8%) | Overall 73, T-ALL 47, T-LBL 26 | Overall 33, T-ALL 24, T-LBL 9 |

Values are n (%) unless otherwise indicated. Intrathecal therapy records were categorized as complete, incomplete, or not assessable. Records were considered incomplete when patients had received treatment at other hospitals before undergoing allo-HSCT at our center and detailed information on those intrathecal administrations was unavailable. Records were considered not assessable when the available information was insufficient to determine whether intrathecal therapy had been administered. The categories of complete, incomplete, and not assessable records were mutually exclusive. For each prophylactic or therapeutic category before or after allo-HSCT, percentages were calculated among patients for whom receipt of that specific category could be determined. Patients with incomplete records were included when at least one corresponding intrathecal administration was documented.

Abbreviations: allo-HSCT, allogeneic hematopoietic stem cell transplantation; CNS, central nervous system; T-ALL, T-cell acute lymphoblastic leukemia; T-LBL, T-cell lymphoblastic lymphoma.

**Table S3. Intrathecal regimens and number of administrations among patients with complete treatment records.**

| **Timing and indication** | **Characteristic** | **Overall** | **T-ALL** | **T-LBL** |
| --- | --- | --- | --- | --- |
| Before allo-HSCT, prophylactic | Recipients with complete records, n | 68 | 43 | 25 |
|  | Regimen(s), n (%) |  |  |  |
|  | Triple intrathecal therapy | 27 (39.7%) | 17 (39.5%) | 10 (40.0%) |
|  | Other regimen(s) | 58 (85.3%) | 36 (83.7%) | 22 (88.0%) |
|  | Administrations per patient, median (range) | 6 (1–12) | 6 (1–8) | 6 (1–12) |
| Before allo-HSCT, therapeutic | Recipients with complete records, n | 4 | 3 | 1 |
|  | Regimen(s), n (%) |  |  |  |
|  | Triple intrathecal therapy | 4 (100.0%) | 3 (100.0%) | 1 (100.0%) |
|  | Other regimen(s) | 2 (50.0%) | 2 (66.7%) | 0 (0.0%) |
|  | Administrations per patient, median (range) | 10.5 (4–12) | 12 (4–12) | 9 (9–9) |
| After allo-HSCT, prophylactic | Recipients with complete records, n | 10 | 5 | 5 |
|  | Regimen(s), n (%) |  |  |  |
|  | Triple intrathecal therapy | 2 (20.0%) | 2 (40.0%) | 0 (0.0%) |
|  | Other regimen(s) | 9 (90.0%) | 4 (80.0%) | 5 (100.0%) |
|  | Administrations per patient, median (range) | 1 (1–5) | 1 (1–5) | 1 (1–1) |
| After allo-HSCT, therapeutic | Recipients with complete records, n | 5 | 4 | 1 |
|  | Regimen(s), n (%) |  |  |  |
|  | Triple intrathecal therapy | 4 (80.0%) | 3 (75.0%) | 1 (100.0%) |
|  | Other regimen(s) | 2 (40.0%) | 2 (50.0%) | 0 (0.0%) |
|  | Administrations per patient, median (range) | 8 (5–13) | 10 (6–13) | 5 (5–5) |

Detailed results for regimens and number of administrations were restricted to confirmed recipients with complete records. Triple intrathecal therapy comprised methotrexate, cytarabine, and a glucocorticoid administered during the same procedure. Other regimens comprised cytarabine plus dexamethasone or methotrexate plus dexamethasone. Regimen categories were not mutually exclusive. No recipient with complete records received methotrexate alone, cytarabine alone, or methotrexate plus cytarabine without a glucocorticoid.

Abbreviations: allo-HSCT, allogeneic hematopoietic stem cell transplantation; T-ALL, T-cell acute lymphoblastic leukemia; T-LBL, T-cell lymphoblastic lymphoma.

**Table S4. Initial NIH 2014 cGVHD severity at first diagnosis.**

| Severity | n | % of NIH cGVHD cases |
| --- | --- | --- |
| Mild | 10 | 47.6% |
| Moderate | 10 | 47.6% |
| Severe | 1 | 4.8% |
| Total | 21 | 100.0% |

Severity was assessed at the first documented diagnosis of cGVHD according to the NIH 2014 consensus criteria. Percentages were calculated among patients with NIH-defined cGVHD (n=21).

Abbreviations: NIH, National Institutes of Health; cGVHD, chronic graft-versus-host disease.

**Table S5. Standardized mean differences before and after propensity score matching.**

| Variable | SMD | | |
| --- | --- | --- | --- |
|  | **All patients before matching**  (N=106) | **2:1 PSM** (N=70) | **1:1 PSM** (N=52) |
| Age at allo-HSCT, years | -0.030 | 0.004 | 0.032 |
| Age group |  |  |  |
| ≤35 | 0.012 | 0.019 | 0.000 |
| >35 | -0.012 | -0.019 | 0.000 |
| Sex |  |  |  |
| Female | -0.039 | -0.038 | -0.038 |
| Male | 0.039 | 0.038 | 0.038 |
| BM blasts at diagnosis, % | -9.449 | -9.997 | -10.505 |
| WBC at diagnosis |  |  |  |
| ≤100 × 10^9^/L | 0.114 | 0.118 | 0.120 |
| >100 × 10^9^/L | -0.114 | -0.118 | -0.120 |
| Missing | — | — | — |
| LDH at diagnosis |  |  |  |
| Normal | 0.007 | 0.068 | 0.105 |
| Elevated | -0.007 | -0.068 | -0.105 |
| Missing | — | — | — |
| CNS involvement at diagnosis |  |  |  |
| No | 0.042 | 0.058 | 0.038 |
| Yes | -0.042 | -0.058 | -0.038 |
| Mediastinal involvement at diagnosis |  |  |  |
| No | -0.172 | -0.142 | -0.157 |
| Yes | 0.172 | 0.142 | 0.157 |
| Missing | — | — | — |
| ETP immunophenotype at diagnosis |  |  |  |
| Non-ETP | 0.203 | 0.201 | 0.273 |
| ETP | -0.203 | -0.201 | -0.273 |
| Missing | — | — | — |
| HCT-CI score used for PSM (0, 1, and ≥2) | 0.310 | -0.088 | -0.234 |
| HCT-CI |  |  |  |
| 0 | -0.160 | 0.038 | 0.115 |
| 1 | 0.117 | -0.019 | -0.077 |
| ≥2 | 0.043 | -0.019 | -0.038 |
| Disease status at allo-HSCT |  |  |  |
| CR1 | -0.188 | -0.019 | -0.038 |
| CR2 | 0.015 | 0.038 | 0.038 |
| Non-CR | 0.172 | -0.019 | 0.000 |
| MRD status before allo-HSCT |  |  |  |
| MRD− | 0.150 | 0.180 | 0.174 |
| MRD+ | -0.150 | -0.180 | -0.174 |
| Cycles of chemotherapy before allo-HSCT | 0.259 | 0.120 | 0.156 |
| Chemotherapy cycles group |  |  |  |
| ≤4 | -0.080 | 0.019 | -0.038 |
| ≥5 | 0.080 | -0.019 | 0.038 |
| Time from diagnosis to allo-HSCT, transformed for PSM | 0.196 | 0.055 | 0.051 |
| Time from diagnosis to allo-HSCT, months | 0.166 | 0.033 | 0.038 |
| Time from diagnosis to allo-HSCT (categorical) |  |  |  |
| <8 months | -0.021 | 0.000 | 0.000 |
| ≥8 months | 0.021 | 0.000 | 0.000 |
| Year of transplantation | -0.276 | -0.027 | -0.053 |
| Transplant era |  |  |  |
| 2010–2018 | 0.149 | 0.019 | 0.077 |
| 2019–2025 | -0.149 | -0.019 | -0.077 |
| Conditioning regimen |  |  |  |
| mBuCy | 0.039 | 0.058 | 0.000 |
| TBI-Cy | -0.082 | -0.096 | -0.038 |
| Others | 0.043 | 0.038 | 0.038 |
| Donor type |  |  |  |
| MSD | -0.110 | -0.038 | 0.000 |
| HID | 0.037 | 0.000 | 0.000 |
| MUD | 0.073 | 0.038 | 0.000 |
| GVHD prophylaxis |  |  |  |
| CSA+MTX+MMF | -0.055 | -0.077 | -0.038 |
| CSA+MTX+MMF+ATG | 0.055 | 0.077 | 0.038 |
| Donor-recipient gender matching |  |  |  |
| Others | 0.109 | 0.077 | 0.077 |
| Female-male | -0.109 | -0.077 | -0.077 |
| MNCs, × 10^8^/kg | -0.015 | 0.156 | 0.153 |
| CD34+ cells, × 10^6^/kg | -0.081 | -0.288 | -0.117 |

Signed SMDs were calculated as T-LBL minus T-ALL. An absolute SMD below 0.10 indicated adequate balance. For HCT-CI and time from diagnosis to allo-HSCT, balance was assessed using the rows identified as being included in the propensity score model. The remaining rows for these variables are provided for descriptive purposes. For variables with missing data, descriptive SMDs were calculated using patients with recorded values for the corresponding variable. Patients with missing values were not excluded from PSM because these variables were not included in the propensity score model.

Abbreviations: SMD, standardized mean difference; PSM, propensity score matching; allo-HSCT, allogeneic hematopoietic stem cell transplantation; BM, bone marrow; WBC, white blood cell; LDH, lactic dehydrogenase; CNS, central nervous system; ETP, early T-cell precursor; HCT-CI, hematopoietic cell transplantation comorbidity index; CR1, first complete remission; CR2, second complete remission; non-CR, non–complete remission; MRD, measurable residual disease; mBuCy, modified busulfan plus cyclophosphamide; TBI-Cy, total body irradiation plus cyclophosphamide; MSD, human leukocyte antigen (HLA)-matched sibling donors; HID, HLA-haploidentical related donors; MUD, HLA-matched unrelated donors; GVHD, graft-versus-host disease; CSA, cyclosporine A; MTX, methotrexate; MMF, mycophenolate mofetil; ATG, antithymocyte globulin; MNC, mononuclear cell; T-ALL, T-cell acute lymphoblastic leukemia; T-LBL, T-cell lymphoblastic lymphoma.

**Table S6. Baseline characteristics of the 2:1 and strict 1:1 PSM cohorts.**

| **Characteristic** | **2:1 PSM cohort** | | | | **Strict 1:1 PSM cohort** | | | |
| --- | --- | --- | --- | --- | --- | --- | --- | --- |
|  | **Total (n = 70)** | **T-ALL (n = 44)** | **T-LBL (n = 26)** | **P value** | **Total (n = 52)** | **T-ALL (n = 26)** | **T-LBL (n = 26)** | **P value** |
| Age at allo-HSCT, years |  |  |  |  |  |  |  |  |
| Median (IQR) | 25.5 (22.0-32.0) | 26.0 (22.0-32.2) | 24.5 (22.0-32.0) | 0.747 | 25.0 (21.8-32.0) | 25.0 (21.2-31.5) | 24.5 (22.0-32.0) | 0.927 |
| ≤ 35 | 58 (82.9%) | 36 (81.8%) | 22 (84.6%) | 1.000 | 44 (84.6%) | 22 (84.6%) | 22 (84.6%) | 1.000 |
| > 35 | 12 (17.1%) | 8 (18.2%) | 4 (15.4%) |  | 8 (15.4%) | 4 (15.4%) | 4 (15.4%) |  |
| Sex, n (%) |  |  |  | 0.557 |  |  |  | 0.749 |
| Female | 19 (27.1%) | 13 (29.5%) | 6 (23.1%) |  | 13 (25.0%) | 7 (26.9%) | 6 (23.1%) |  |
| Male | 51 (72.9%) | 31 (70.5%) | 20 (76.9%) |  | 39 (75.0%) | 19 (73.1%) | 20 (76.9%) |  |
| BM blasts at diagnosis, %, median (IQR) | 58.5 (6.9-89.8) | 87.5 (69.7-94.4) | 1.4 (0.4-8.9) | <0.001 | 26.5 (1.5-88.4) | 88.4 (80.4-94.3) | 1.4 (0.4-8.9) | <0.001 |
| WBC at diagnosis, n (%) |  |  |  | 0.149 |  |  |  | 0.110 |
| ≤100 × 10⁹/L | 64 (91.4%) | 38 (86.4%) | 26 (100.0%) |  | 48 (92.3%) | 22 (84.6%) | 26 (100.0%) |  |
| >100 × 10⁹/L | 5 (7.1%) | 5 (11.4%) | 0 (0.0%) |  | 3 (5.8%) | 3 (11.5%) | 0 (0.0%) |  |
| Missing | 1 (1.4%) | 1 (2.3%) | 0 (0.0%) |  | 1 (1.9%) | 1 (3.8%) | 0 (0.0%) |  |
| LDH at diagnosis, n (%) |  |  |  | 0.869 |  |  |  | 0.491 |
| Normal | 26 (37.1%) | 16 (36.4%) | 10 (38.5%) |  | 17 (32.7%) | 7 (26.9%) | 10 (38.5%) |  |
| Elevated | 33 (47.1%) | 21 (47.7%) | 12 (46.2%) |  | 25 (48.1%) | 13 (50.0%) | 12 (46.2%) |  |
| Missing | 11 (15.7%) | 7 (15.9%) | 4 (15.4%) |  | 10 (19.2%) | 6 (23.1%) | 4 (15.4%) |  |
| CNS involvement at diagnosis, n (%) |  |  |  | 0.526 |  |  |  | 1.000 |
| No | 68 (97.1%) | 42 (95.5%) | 26 (100.0%) |  | 51 (98.1%) | 25 (96.2%) | 26 (100.0%) |  |
| Yes | 2 (2.9%) | 2 (4.5%) | 0 (0.0%) |  | 1 (1.9%) | 1 (3.8%) | 0 (0.0%) |  |
| Mediastinal involvement at diagnosis, n (%) |  |  |  | 0.183 |  |  |  | 0.260 |
| No | 42 (60.0%) | 28 (63.6%) | 14 (53.8%) |  | 30 (57.7%) | 16 (61.5%) | 14 (53.8%) |  |
| Yes | 24 (34.3%) | 12 (27.3%) | 12 (46.2%) |  | 19 (36.5%) | 7 (26.9%) | 12 (46.2%) |  |
| Missing | 4 (5.7%) | 4 (9.1%) | 0 (0.0%) |  | 3 (5.8%) | 3 (11.5%) | 0 (0.0%) |  |
| ETP immunophenotype at diagnosis, n (%) |  |  |  | 0.059 |  |  |  | 0.038 |
| Non-ETP | 45 (64.3%) | 25 (56.8%) | 20 (76.9%) |  | 34 (65.4%) | 14 (53.8%) | 20 (76.9%) |  |
| ETP | 20 (28.6%) | 16 (36.4%) | 4 (15.4%) |  | 15 (28.8%) | 11 (42.3%) | 4 (15.4%) |  |
| Missing | 5 (7.1%) | 3 (6.8%) | 2 (7.7%) |  | 3 (5.8%) | 1 (3.8%) | 2 (7.7%) |  |
| HCT-CI, n (%) |  |  |  | 1.000 |  |  |  | 0.647 |
| 0 | 51 (72.9%) | 32 (72.7%) | 19 (73.1%) |  | 35 (67.3%) | 16 (61.5%) | 19 (73.1%) |  |
| 1 | 16 (22.9%) | 10 (22.7%) | 6 (23.1%) |  | 14 (26.9%) | 8 (30.8%) | 6 (23.1%) |  |
| ≥ 2 | 3 (4.3%) | 2 (4.5%) | 1 (3.8%) |  | 3 (5.8%) | 2 (7.7%) | 1 (3.8%) |  |
| Disease status at allo-HSCT, n (%) |  |  |  | 0.655 |  |  |  | 1.000 |
| CR1 | 60 (85.7%) | 39 (88.6%) | 21 (80.8%) |  | 43 (82.7%) | 22 (84.6%) | 21 (80.8%) |  |
| CR2 | 3 (4.3%) | 1 (2.3%) | 2 (7.7%) |  | 3 (5.8%) | 1 (3.8%) | 2 (7.7%) |  |
| Non-CR | 7 (10.0%) | 4 (9.1%) | 3 (11.5%) |  | 6 (11.5%) | 3 (11.5%) | 3 (11.5%) |  |
| MRD status before allo-HSCT, n (%) |  |  |  | 0.301 |  |  |  | 0.243 |
| MRD− | 52 (82.5%) | 31 (77.5%) | 21 (91.3%) |  | 38 (82.6%) | 17 (73.9%) | 21 (91.3%) |  |
| MRD+ | 11 (17.5%) | 9 (22.5%) | 2 (8.7%) |  | 8 (17.4%) | 6 (26.1%) | 2 (8.7%) |  |
| Cycles of chemotherapy before allo-HSCT |  |  |  |  |  |  |  |  |
| Median (IQR) | 5.0 (4.0-6.0) | 5.0 (4.0-5.0) | 5.0 (4.0-6.0) | 0.416 | 5.0 (4.0-6.0) | 5.0 (4.0-5.0) | 5.0 (4.0-6.0) | 0.283 |
| ≤ 4 | 23 (32.9%) | 14 (31.8%) | 9 (34.6%) | 0.810 | 19 (36.5%) | 10 (38.5%) | 9 (34.6%) | 0.773 |
| ≥ 5 | 47 (67.1%) | 30 (68.2%) | 17 (65.4%) |  | 33 (63.5%) | 16 (61.5%) | 17 (65.4%) |  |
| Time from diagnosis to allo-HSCT, months |  |  |  |  |  |  |  |  |
| Median (IQR) | 7.0 (6.0-8.0) | 7.0 (6.0-8.2) | 7.0 (6.0-8.0) | 0.917 | 7.0 (6.0-8.0) | 7.0 (6.0-8.0) | 7.0 (6.0-8.0) | 0.978 |
| < 8 | 43 (61.4%) | 27 (61.4%) | 16 (61.5%) | 0.988 | 32 (61.5%) | 16 (61.5%) | 16 (61.5%) | 1.000 |
| ≥ 8 | 27 (38.6%) | 17 (38.6%) | 10 (38.5%) |  | 20 (38.5%) | 10 (38.5%) | 10 (38.5%) |  |
| Year of transplantation |  |  |  |  |  |  |  |  |
| Median (IQR) | 2018 (2015-2020) | 2018 (2015-2020) | 2018 (2014-2020) | 0.864 | 2018 (2015-2020) | 2019 (2015-2020) | 2018 (2014-2020) | 0.993 |
| ≤ 2018 | 38 (54.3%) | 24 (54.5%) | 14 (53.8%) | 0.955 | 26 (50.0%) | 12 (46.2%) | 14 (53.8%) | 0.579 |
| > 2018 | 32 (45.7%) | 20 (45.5%) | 12 (46.2%) |  | 26 (50.0%) | 14 (53.8%) | 12 (46.2%) |  |
| Conditioning regimen, n (%) |  |  |  | 0.370 |  |  |  | 1.000 |
| mBuCy | 52 (74.3%) | 32 (72.7%) | 20 (76.9%) |  | 40 (76.9%) | 20 (76.9%) | 20 (76.9%) |  |
| TBI-Cy | 17 (24.3%) | 12 (27.3%) | 5 (19.2%) |  | 11 (21.2%) | 6 (23.1%) | 5 (19.2%) |  |
| Others | 1 (1.4%) | 0 (0.0%) | 1 (3.8%) |  | 1 (1.9%) | 0 (0.0%) | 1 (3.8%) |  |
| Donor type, n (%) |  |  |  | 0.743 |  |  |  | 1.000 |
| MSD | 19 (27.1%) | 13 (29.5%) | 6 (23.1%) |  | 12 (23.1%) | 6 (23.1%) | 6 (23.1%) |  |
| HID | 40 (57.1%) | 25 (56.8%) | 15 (57.7%) |  | 30 (57.7%) | 15 (57.7%) | 15 (57.7%) |  |
| MUD | 11 (15.7%) | 6 (13.6%) | 5 (19.2%) |  | 10 (19.2%) | 5 (19.2%) | 5 (19.2%) |  |
| GVHD prophylaxis, n (%) |  |  |  | 0.514 |  |  |  | 1.000 |
| CSA+MTX+MMF | 12 (17.1%) | 9 (20.5%) | 3 (11.5%) |  | 7 (13.5%) | 4 (15.4%) | 3 (11.5%) |  |
| CSA+MTX+MMF+ATG | 58 (82.9%) | 35 (79.5%) | 23 (88.5%) |  | 45 (86.5%) | 22 (84.6%) | 23 (88.5%) |  |
| Donor-recipient gender matching, n (%) |  |  |  | 0.331 |  |  |  | 0.532 |
| Female–male | 21 (30.0%) | 15 (34.1%) | 6 (23.1%) |  | 14 (26.9%) | 8 (30.8%) | 6 (23.1%) |  |
| Others | 49 (70.0%) | 29 (65.9%) | 20 (76.9%) |  | 38 (73.1%) | 18 (69.2%) | 20 (76.9%) |  |
| PBSC graft, median (IQR) |  |  |  |  |  |  |  |  |
| MNCs × 10⁸/kg | 8.7 (7.3-11.6) | 8.8 (7.7-11.3) | 8.4 (6.7-12.8) | 0.923 | 8.9 (7.4-11.5) | 9.4 (8.1-11.2) | 8.4 (6.7-12.8) | 0.735 |
| CD34+ cells × 10⁶/kg | 4.2 (3.1-5.4) | 4.4 (3.2-5.8) | 3.97 (3.1-5.3) | 0.412 | 4.1 (3.2-5.3) | 4.4 (3.3-5.3) | 4.0 (3.1-5.3) | 0.492 |

MRD status was evaluated only in patients who were in complete remission at the time of allo-HSCT, and percentages were calculated using these evaluable patients as the denominator.

Abbreviations: PSM, propensity score matching; SMD, standardized mean difference; T-ALL, T-cell acute lymphoblastic leukemia; T-LBL, T-cell lymphoblastic lymphoma; allo-HSCT, allogeneic hematopoietic stem cell transplantation; IQR, interquartile range; BM, bone marrow; WBC, white blood cell; LDH, lactic dehydrogenase; CNS, central nervous system; ETP, early T-cell precursor; HCT-CI, hematopoietic cell transplantation comorbidity index; CR1, first complete remission; CR2, second complete remission; non-CR, non-complete remission; MRD, measurable residual disease; mBuCy, modified busulfan plus cyclophosphamide; TBI-Cy, total body irradiation plus cyclophosphamide; MSD, human leukocyte antigen (HLA)-matched sibling donors; HID, HLA-haploidentical related donors; MUD, HLA-matched unrelated donors; GVHD, graft-versus-host disease; CSA, cyclosporine A; MMF, mycophenolate mofetil; MTX, methotrexate; ATG, antithymocyte globulin; MNCs, mononuclear cells.

**Table S7. Outcomes in the 2:1 and strict 1:1 PSM cohorts and the CR1 subgroup.**

| **Outcome** | **Time point** | **2:1 PSM cohort** | | | **Strict 1:1 PSM cohort** | | | **CR1 subgroup** | | |
| --- | --- | --- | --- | --- | --- | --- | --- | --- | --- | --- |
|  |  | T-**ALL**   **(n=44)** | **T-LBL**  **(n=26)** | **P value** | T-**ALL**   **(n=26)** | **T-LBL**  **(n=26)** | **P value** | **T-ALL**  **(n=62)** | **T-LBL**  **(n=24)** | **P value** |
| OS | 1 year | 75.8% (59.7–86.2) | 65.4% (44.0–80.3) | 0.611 | 79.7% (57.9–91.0) | 65.4% (44.0–80.3) | 0.879 | 77.0% (64.2–85.7) | 66.7% (44.3–81.7) | 0.454 |
|  | 2 years | 57.7% (41.0–71.3) | 53.6% (32.9–70.4) |  | 54.6% (33.1–71.8) | 53.6% (32.9–70.4) |  | 62.8% (49.1–73.8) | 49.7% (28.7–67.6) |  |
|  | 3 years | 54.7% (37.9–68.7) | 49.1% (28.8–66.6) |  | 49.6% (28.4–67.7) | 49.1% (28.8–66.6) |  | 56.8% (42.9–68.5) | 49.7% (28.7–67.6) |  |
|  | 5 years | 51.5% (34.7–66.0) | 49.1% (28.8–66.6) |  | 44.1% (23.4–63.1) | 49.1% (28.8–66.6) |  | 52.2% (38.1–64.5) | 49.7% (28.7–67.6) |  |
| DFS | 1 year | 66.3% (49.7–78.5) | 57.7% (36.8–73.9) | 0.796 | 68.0% (46.0–82.6) | 57.7% (36.8–73.9) | 0.937 | 67.2% (53.9–77.5) | 58.3% (36.4–75.0) | 0.615 |
|  | 2 years | 53.2% (36.7–67.2) | 50.0% (29.9–67.2) |  | 51.0% (30.0–68.6) | 50.0% (29.9–67.2) |  | 56.5% (43.0–68.0) | 50.0% (29.1–67.8) |  |
|  | 3 years | 50.2% (33.8–64.6) | 50.0% (29.9–67.2) |  | 46.4% (25.9–64.6) | 50.0% (29.9–67.2) |  | 54.4% (40.8–66.2) | 50.0% (29.1–67.8) |  |
|  | 5 years | 47.1% (30.8–61.8) | 50.0% (29.9–67.2) |  | 41.2% (21.4–60.1) | 50.0% (29.9–67.2) |  | 49.9% (36.1–62.2) | 50.0% (29.1–67.8) |  |
| NRM | 1 year | 9.6% (3.7–24.0) | 15.4% (5.9–36.6) | 0.879 | 3.8% (0.5–25.2) | 15.4% (5.9–36.6) | 0.978 | 9.9% (4.5–20.8) | 20.8% (9.1–43.7) | 0.335 |
|  | 2 years | 15.0% (6.9–30.7) | 15.4% (5.9–36.6) |  | 12.3% (4.0–34.6) | 15.4% (5.9–36.6) |  | 15.3% (8.2–27.5) | 25.0% (11.9–48.1) |  |
|  | 3 years | 17.9% (8.8–34.5) | 15.4% (5.9–36.6) |  | 17.0% (6.5–40.4) | 15.4% (5.9–36.6) |  | 17.4% (9.6–30.2) | 25.0% (11.9–48.1) |  |
|  | 5 years | 17.9% (8.8–34.5) | 15.4% (5.9–36.6) |  | 17.0% (6.5–40.4) | 15.4% (5.9–36.6) |  | 17.4% (9.6–30.2) | 25.0% (11.9–48.1) |  |
| Relapse | 1 year | 24.1% (13.6–40.5) | 26.9% (13.6–48.9) | 0.840 | 28.1% (14.2–50.9) | 26.9% (13.6–48.9) | 0.790 | 22.9% (14.2–35.8) | 20.8% (9.1–43.7) | 0.643 |
|  | 2 years | 31.9% (19.7–48.9) | 34.6% (19.5–56.6) |  | 36.6% (20.6–59.5) | 34.6% (19.5–56.6) |  | 28.2% (18.5–41.6) | 25.0% (11.9–48.1) |  |
|  | 3 years | 31.9% (19.7–48.9) | 34.6% (19.5–56.6) |  | 36.6% (20.6–59.5) | 34.6% (19.5–56.6) |  | 28.2% (18.5–41.6) | 25.0% (11.9–48.1) |  |
|  | 5 years | 35.0% (22.1–52.4) | 34.6% (19.5–56.6) |  | 41.8% (24.4–64.9) | 34.6% (19.5–56.6) |  | 32.7% (22.0–46.8) | 25.0% (11.9–48.1) |  |
| Grade II–IV aGVHD | 30 days | 22.7% (10.2–35.3) | 19.2% (3.8–34.7) | 0.456 | 23.1% (6.5–39.6) | 19.2% (3.8–34.7) | 0.465 | 21.0% (10.7–31.2) | 25.0% (7.3–42.7) | 0.552 |
|  | 60 days | 29.5% (15.9–43.2) | 30.8% (12.6–48.9) |  | 26.9% (9.5–44.4) | 30.8% (12.6–48.9) |  | 30.6% (19.1–42.2) | 33.3% (14.0–52.7) |  |
|  | 100 days | 40.9% (26.2–55.7) | 30.8% (12.6–48.9) |  | 42.3% (22.8–61.8) | 30.8% (12.6–48.9) |  | 41.9% (29.5–54.4) | 33.3% (14.0–52.7) |  |
| Grade III–IV aGVHD | 30 days | 6.8% (0.0–14.4) | 0.0% (0.0–0.0) | 0.618 | 3.8% (0.0–11.4) | 0.0% (0.0–0.0) | 1.000 | 4.8% (0.0–10.2) | 0.0% (0.0–0.0) | 0.236 |
|  | 60 days | 6.8% (0.0–14.4) | 7.7% (0.0–18.1) |  | 3.8% (0.0–11.4) | 7.7% (0.0–18.1) |  | 8.1% (1.2–14.9) | 4.2% (0.0–12.3) |  |
|  | 100 days | 11.4% (1.9–20.9) | 7.7% (0.0–18.1) |  | 7.7% (0.0–18.1) | 7.7% (0.0–18.1) |  | 12.9% (4.5–21.3) | 4.2% (0.0–12.3) |  |
| Any NIH cGVHD | 1 year | 23.5% (10.6–36.4) | 3.8% (0.0–11.4) | 0.052 | 23.3% (6.6–40.0) | 3.8% (0.0–11.4) | 0.052 | 22.8% (12.2–33.3) | 4.2% (0.0–12.4) | 0.061 |
|  | 2 years | 26.3% (12.7–39.9) | 7.7% (0.0–18.2) |  | 27.7% (9.7–45.7) | 7.7% (0.0–18.2) |  | 26.6% (15.3–37.9) | 8.3% (0.0–19.8) |  |
|  | 3 years | 26.3% (12.7–39.9) | 7.7% (0.0–18.2) |  | 27.7% (9.7–45.7) | 7.7% (0.0–18.2) |  | 26.6% (15.3–37.9) | 8.3% (0.0–19.8) |  |
|  | 5 years | 26.3% (12.7–39.9) | 7.7% (0.0–18.2) |  | 27.7% (9.7–45.7) | 7.7% (0.0–18.2) |  | 26.6% (15.3–37.9) | 8.3% (0.0–19.8) |  |
| Moderate–severe cGVHD | 1 year | 16.4% (5.2–27.7) | 0.0% (0.0–0.0) | 0.031 | 15.6% (1.2–30.0) | 0.0% (0.0–0.0) | 0.038 | 14.7% (5.8–23.6) | 0.0% (0.0–0.0) | 0.035 |
|  | 2 years | 16.4% (5.2–27.7) | 0.0% (0.0–0.0) |  | 15.6% (1.2–30.0) | 0.0% (0.0–0.0) |  | 16.6% (7.1–26.1) | 0.0% (0.0–0.0) |  |
|  | 3 years | 16.4% (5.2–27.7) | 0.0% (0.0–0.0) |  | 15.6% (1.2–30.0) | 0.0% (0.0–0.0) |  | 16.6% (7.1–26.1) | 0.0% (0.0–0.0) |  |
|  | 5 years | 16.4% (5.2–27.7) | 0.0% (0.0–0.0) |  | 15.6% (1.2–30.0) | 0.0% (0.0–0.0) |  | 16.6% (7.1–26.1) | 0.0% (0.0–0.0) |  |
| GRFS | 1 year | 46.6% (31.2–60.6) | 50.0% (29.9–67.2) | 0.481 | 48.6% (28.2–66.3) | 50.0% (29.9–67.2) | 0.414 | 47.9% (34.9–59.7) | 50.0% (29.1–67.8) | 0.664 |
|  | 2 years | 30.4% (17.0–44.9) | 38.5% (20.4–56.3) |  | 26.5% (11.0–45.0) | 38.5% (20.4–56.3) |  | 34.7% (22.8–47.0) | 37.5% (19.0–56.0) |  |
|  | 3 years | 30.4% (17.0–44.9) | 38.5% (20.4–56.3) |  | 26.5% (11.0–45.0) | 38.5% (20.4–56.3) |  | 34.7% (22.8–47.0) | 37.5% (19.0–56.0) |  |
|  | 5 years | 27.6% (14.8–42.0) | 38.5% (20.4–56.3) |  | 22.1% (8.2–40.2) | 38.5% (20.4–56.3) |  | 30.4% (18.9–42.7) | 37.5% (19.0–56.0) |  |

OS, DFS and GRFS were estimated using Kaplan-Meier methods and compared using log-rank tests. CIR, NRM and GVHD outcomes were estimated using cumulative incidence functions and compared using Gray tests. The CR1 subgroup was not propensity score matched. Values are percentages (95% CIs).

Abbreviations: OS, overall survival; DFS, disease-free survival; NRM, non-relapse mortality; GRFS, graft-versus-host disease-free, relapse-free survival; aGVHD, acute graft-versus-host disease; NIH, National Institutes of Health; cGVHD, chronic graft-versus-host disease; PSM, propensity score matching; CR1, first complete remission; T-ALL, T-cell acute lymphoblastic leukemia; T-LBL, T-cell lymphoblastic lymphoma; CIR, cumulative incidence of relapse; CI, confidence interval.

**Table S8. Univariable analysis for clinical outcomes in all patients.**

| **Characteristic** | **OS** | | **DFS** | | **GRFS** | | **NRM** | | **CIR** | |
| --- | --- | --- | --- | --- | --- | --- | --- | --- | --- | --- |
|  | **HR (95% CI)** | **P value** | **HR (95% CI)** | **P value** | **HR (95% CI)** | **P value** | **sHR (95% CI)** | **P value** | **sHR (95% CI)** | **P value** |
| Sex, n (%) |  |  |  |  |  |  |  |  |  |  |
| Male | 1.00 (ref.) |  | 1.00 (ref.) |  | 1.00 (ref.) |  | 1.00 (ref.) |  | 1.00 (ref.) |  |
| Female | 0.44 (0.21–0.90) | 0.024 | 0.52 (0.27–1.02) | 0.056 | 0.66 (0.39–1.15) | 0.141 | 0.29 (0.07–1.25) | 0.097 | 0.77 (0.38–1.59) | 0.482 |
| Diagnosis, n (%) |  |  |  |  |  |  |  |  |  |  |
| T-ALL | 1.00 (ref.) |  | 1.00 (ref.) |  | 1.00 (ref.) |  | 1.00 (ref.) |  | 1.00 (ref.) |  |
| T-LBL | 1.59 (0.90–2.79) | 0.108 | 1.42 (0.82–2.47) | 0.210 | 0.96 (0.58–1.57) | 0.856 | 1.32 (0.51–3.44) | 0.565 | 1.22 (0.61–2.41) | 0.575 |
| Age at allo-HSCT, years |  |  |  |  |  |  |  |  |  |  |
| ≤35 | 1.00 (ref.) |  | 1.00 (ref.) |  | 1.00 (ref.) |  | 1.00 (ref.) |  | 1.00 (ref.) |  |
| >35 | 1.10 (0.54–2.27) | 0.793 | 1.12 (0.56–2.23) | 0.745 | 1.15 (0.63–2.10) | 0.644 | 0.62 (0.15–2.64) | 0.519 | 1.46 (0.71–2.99) | 0.299 |
| BM blasts at diagnosis, per 10 percentage-point increase | 0.96 (0.89–1.04) | 0.351 | 0.97 (0.90–1.05) | 0.480 | 1.01 (0.95–1.08) | 0.665 | 1.01 (0.89–1.15) | 0.878 | 0.97 (0.89–1.07) | 0.565 |
| WBC at diagnosis, ×10⁹/L |  |  |  |  |  |  |  |  |  |  |
| ≤100 | 1.00 (ref.) |  | 1.00 (ref.) |  | 1.00 (ref.) |  | 1.00 (ref.) |  | 1.00 (ref.) |  |
| >100 | 0.64 (0.20–2.05) | 0.448 | 0.90 (0.32–2.49) | 0.834 | 0.79 (0.32–1.98) | 0.621 | Not estimable | — | 1.60 (0.62–4.08) | 0.328 |
| LDH at diagnosis |  |  |  |  |  |  |  |  |  |  |
| Normal | 1.00 (ref.) |  | 1.00 (ref.) |  | 1.00 (ref.) |  | 1.00 (ref.) |  | 1.00 (ref.) |  |
| Elevated | 1.30 (0.73–2.34) | 0.374 | 1.46 (0.83–2.57) | 0.191 | 1.14 (0.69–1.86) | 0.612 | 0.53 (0.19–1.43) | 0.210 | 2.26 (1.13–4.50) | 0.021 |
| CNS involvement |  |  |  |  |  |  |  |  |  |  |
| No | 1.00 (ref.) |  | 1.00 (ref.) |  | 1.00 (ref.) |  | 1.00 (ref.) |  | 1.00 (ref.) |  |
| Yes | 2.68 (0.65–11.09) | 0.174 | 2.99 (0.72–12.37) | 0.131 | 2.71 (0.85–8.66) | 0.092 | Not estimable | — | 4.66 (1.90–11.42) | <0.001 |
| Mediastinal involvement |  |  |  |  |  |  |  |  |  |  |
| No | 1.00 (ref.) |  | 1.00 (ref.) |  | 1.00 (ref.) |  | 1.00 (ref.) |  | 1.00 (ref.) |  |
| Yes | 1.38 (0.78–2.44) | 0.267 | 1.58 (0.91–2.74) | 0.102 | 1.49 (0.92–2.40) | 0.106 | 0.48 (0.16–1.47) | 0.199 | 2.38 (1.25–4.56) | 0.009 |
| ETP immunophenotype |  |  |  |  |  |  |  |  |  |  |
| Non-ETP | 1.00 (ref.) |  | 1.00 (ref.) |  | 1.00 (ref.) |  | 1.00 (ref.) |  | 1.00 (ref.) |  |
| ETP | 1.08 (0.59–1.97) | 0.799 | 0.88 (0.49–1.59) | 0.680 | 1.03 (0.62–1.70) | 0.918 | 1.51 (0.57–4.00) | 0.410 | 0.71 (0.35–1.43) | 0.339 |
| GVHD prophylaxis |  |  |  |  |  |  |  |  |  |  |
| CSA+MTX+MMF | 1.00 (ref.) |  | 1.00 (ref.) |  | 1.00 (ref.) |  | 1.00 (ref.) |  | 1.00 (ref.) |  |
| CSA+MTX+MMF+ATG | 1.10 (0.49–2.44) | 0.819 | 1.21 (0.55–2.67) | 0.643 | 0.53 (0.29–0.96) | 0.037 | 3.11 (0.46–21.19) | 0.245 | 0.79 (0.34–1.85) | 0.593 |
| MRD status before allo-HSCT |  |  |  |  |  |  |  |  |  |  |
| MRD+ | 1.00 (ref.) |  | 1.00 (ref.) |  | 1.00 (ref.) |  | 1.00 (ref.) |  | 1.00 (ref.) |  |
| MRD− | 0.86 (0.42–1.80) | 0.696 | 0.82 (0.41–1.65) | 0.576 | 0.56 (0.31–1.01) | 0.053 | 1.11 (0.33–3.81) | 0.863 | 0.72 (0.33–1.59) | 0.419 |
| Disease status at allo-HSCT |  |  |  |  |  |  |  |  |  |  |
| CR1 | 1.00 (ref.) |  | 1.00 (ref.) |  | 1.00 (ref.) |  | 1.00 (ref.) |  | 1.00 (ref.) |  |
| CR2 | 2.81 (1.17–6.72) | 0.020 | 3.33 (1.48–7.47) | 0.004 | 2.50 (1.18–5.26) | 0.016 | Not estimable | — | 3.12 (1.17–8.29) | 0.022 |
| Non-CR | 1.43 (0.60–3.38) | 0.416 | 1.33 (0.57–3.14) | 0.510 | 1.29 (0.62–2.71) | 0.498 | Not estimable | — | 2.38 (1.01–5.57) | 0.046 |
| HCT-CI |  |  |  |  |  |  |  |  |  |  |
| 0 | 1.00 (ref.) |  | 1.00 (ref.) |  | 1.00 (ref.) |  | 1.00 (ref.) |  | 1.00 (ref.) |  |
| 1 | 1.34 (0.69–2.59) | 0.381 | 1.42 (0.75–2.68) | 0.281 | 1.73 (1.01–2.99) | 0.048 | 0.73 (0.21–2.54) | 0.626 | 1.75 (0.86–3.58) | 0.125 |
| ≥2 | 3.43 (1.32–8.96) | 0.012 | 3.53 (1.37–9.10) | 0.009 | 3.71 (1.56–8.84) | 0.003 | 0.98 (0.12–8.29) | 0.983 | 3.38 (1.19–9.65) | 0.023 |
| Chemotherapy cycles before allo-HSCT |  |  |  |  |  |  |  |  |  |  |
| ≤4 | 1.00 (ref.) |  | 1.00 (ref.) |  | 1.00 (ref.) |  | 1.00 (ref.) |  | 1.00 (ref.) |  |
| ≥5 | 1.30 (0.72–2.35) | 0.381 | 1.20 (0.68–2.13) | 0.533 | 1.10 (0.67–1.80) | 0.715 | 1.98 (0.65–6.02) | 0.228 | 0.94 (0.49–1.83) | 0.865 |
| Donor type |  |  |  |  |  |  |  |  |  |  |
| MSD | 1.00 (ref.) |  | 1.00 (ref.) |  | 1.00 (ref.) |  | 1.00 (ref.) |  | 1.00 (ref.) |  |
| HID | 1.52 (0.79–2.93) | 0.211 | 1.42 (0.75–2.67) | 0.279 | 0.70 (0.42–1.16) | 0.166 | 7.53 (1.04–54.63) | 0.046 | 0.77 (0.39–1.53) | 0.460 |
| MUD | 0.73 (0.24–2.27) | 0.587 | 0.66 (0.21–2.01) | 0.461 | 0.58 (0.25–1.36) | 0.211 | 5.13 (0.48–54.62) | 0.175 | 0.32 (0.08–1.38) | 0.128 |
| Donor-recipient gender matching |  |  |  |  |  |  |  |  |  |  |
| Others | 1.00 (ref.) |  | 1.00 (ref.) |  | 1.00 (ref.) |  | 1.00 (ref.) |  | 1.00 (ref.) |  |
| Female-male | 0.71 (0.38–1.33) | 0.280 | 0.77 (0.42–1.41) | 0.397 | 0.75 (0.45–1.25) | 0.267 | 0.63 (0.21–1.87) | 0.407 | 0.91 (0.44–1.88) | 0.807 |
| Conditioning regimen |  |  |  |  |  |  |  |  |  |  |
| mBuCy | 1.00 (ref.) |  | 1.00 (ref.) |  | 1.00 (ref.) |  | 1.00 (ref.) |  | 1.00 (ref.) |  |
| TBI-Cy | 0.73 (0.34–1.56) | 0.412 | 0.69 (0.32–1.47) | 0.331 | 0.82 (0.44–1.53) | 0.532 | 0.52 (0.13–2.16) | 0.367 | 0.87 (0.36–2.10) | 0.759 |
| Others | 2.59 (0.91–7.33) | 0.074 | 3.09 (1.21–7.87) | 0.018 | 1.73 (0.69–4.34) | 0.241 | 2.10 (0.50–8.70) | 0.309 | 2.14 (0.54–8.45) | 0.279 |
| Time from diagnosis to allo-HSCT |  |  |  |  |  |  |  |  |  |  |
| <8 months | 1.00 (ref.) |  | 1.00 (ref.) |  | 1.00 (ref.) |  | 1.00 (ref.) |  | 1.00 (ref.) |  |
| ≥8 months | 1.08 (0.62–1.88) | 0.776 | 1.18 (0.69–2.01) | 0.550 | 1.52 (0.95–2.42) | 0.078 | 2.07 (0.81–5.32) | 0.129 | 0.85 (0.45–1.62) | 0.628 |
| Transplant era |  |  |  |  |  |  |  |  |  |  |
| 2010–2018 | 1.00 (ref.) |  | 1.00 (ref.) |  | 1.00 (ref.) |  | 1.00 (ref.) |  | 1.00 (ref.) |  |
| 2019–2025 | 0.94 (0.54–1.63) | 0.826 | 1.03 (0.60–1.76) | 0.918 | 0.94 (0.59–1.49) | 0.780 | 0.47 (0.18–1.23) | 0.125 | 1.54 (0.80–2.95) | 0.196 |
| MNC dose, per 1 ×10⁸/kg | 0.99 (0.92–1.07) | 0.817 | 1.02 (0.95–1.09) | 0.662 | 0.99 (0.93–1.06) | 0.815 | 0.95 (0.88–1.04) | 0.287 | 1.05 (0.96–1.14) | 0.260 |
| CD34+ cell dose, per 1 ×10⁶/kg | 1.06 (0.97–1.15) | 0.203 | 1.04 (0.96–1.13) | 0.294 | 1.03 (0.96–1.10) | 0.454 | 0.99 (0.88–1.13) | 0.936 | 1.05 (0.97–1.14) | 0.221 |

HRs are reported for OS, DFS, and GRFS. sHRs are reported for CIR and NRM. Analyses included patients with recorded values for each variable. MRD analyses were restricted to patients in complete remission with evaluable MRD. Not estimable indicates that the coefficient could not be estimated because of sparse or zero target events.

Abbreviations: OS, overall survival; DFS, disease-free survival; GRFS, graft-versus-host disease-free, relapse-free survival; NRM, non-relapse mortality; CIR, cumulative incidence of relapse; HR, hazard ratio; sHR, subdistribution hazard ratio; CI, confidence interval; T-ALL, T-cell acute lymphoblastic leukemia; T-LBL, T-cell lymphoblastic lymphoma; allo-HSCT, allogeneic hematopoietic stem cell transplantation; BM, bone marrow; WBC, white blood cell; LDH, lactic dehydrogenase; CNS, central nervous system; ETP, early T-cell precursor; GVHD, graft-versus-host disease; CSA, cyclosporine A; MMF, mycophenolate mofetil; MTX, methotrexate; ATG, antithymocyte globulin; MRD, measurable residual disease; CR1, first complete remission; CR2, second complete remission; non-CR, non-complete remission; HCT-CI, hematopoietic cell transplantation comorbidity index; MSD, human leukocyte antigen (HLA)-matched sibling donors; HID, HLA-haploidentical related donors; MUD, HLA-matched unrelated donors; mBuCy, modified busulfan plus cyclophosphamide; TBI-Cy, total body irradiation plus cyclophosphamide; MNCs, mononuclear cells.

**Table S9. Univariable analysis for GVHD in all patients.**

| **Characteristic** | **Grade II-IV aGVHD** | | **Grade III-IV aGVHD** | | **Any NIH cGVHD** | |
| --- | --- | --- | --- | --- | --- | --- |
|  | **sHR (95% CI)** | **P value** | **sHR (95% CI)** | **P value** | **sHR (95% CI)** | **P value** |
| Sex, n (%) |  |  |  |  |  |  |
| Male | 1.00 (ref.) |  | 1.00 (ref.) |  | 1.00 (ref.) |  |
| Female | 0.40 (0.17–0.94) | 0.037 | 0.54 (0.12–2.45) | 0.427 | 1.62 (0.69–3.81) | 0.269 |
| Diagnosis, n (%) |  |  |  |  |  |  |
| T-ALL | 1.00 (ref.) |  | 1.00 (ref.) |  | 1.00 (ref.) |  |
| T-LBL | 0.67 (0.33–1.39) | 0.285 | 0.43 (0.10–1.98) | 0.282 | 0.30 (0.09–0.99) | 0.047 |
| Age at allo-HSCT, years |  |  |  |  |  |  |
| ≤35 | 1.00 (ref.) |  | 1.00 (ref.) |  | 1.00 (ref.) |  |
| >35 | 0.80 (0.34–1.89) | 0.611 | 2.82 (0.84–9.47) | 0.094 | 0.84 (0.25–2.82) | 0.772 |
| BM blasts at diagnosis, per 10 percentage-point increase | 1.07 (0.98–1.18) | 0.135 | 1.15 (0.94–1.41) | 0.177 | 1.11 (0.98–1.25) | 0.096 |
| WBC at diagnosis, ×10⁹/L |  |  |  |  |  |  |
| ≤100 | 1.00 (ref.) |  | 1.00 (ref.) |  | 1.00 (ref.) |  |
| >100 | 1.53 (0.55–4.28) | 0.414 | 1.26 (0.15–10.33) | 0.827 | Not estimable | — |
| LDH at diagnosis |  |  |  |  |  |  |
| Normal | 1.00 (ref.) |  | 1.00 (ref.) |  | 1.00 (ref.) |  |
| Elevated | 1.56 (0.80–3.02) | 0.190 | 0.76 (0.24–2.45) | 0.646 | 0.49 (0.19–1.31) | 0.157 |
| CNS involvement |  |  |  |  |  |  |
| No | 1.00 (ref.) |  | 1.00 (ref.) |  | 1.00 (ref.) |  |
| Yes | 1.96 (0.63–6.04) | 0.242 | 4.21 (0.51–34.73) | 0.182 | 2.56 (0.54–12.14) | 0.237 |
| Mediastinal involvement |  |  |  |  |  |  |
| No | 1.00 (ref.) |  | 1.00 (ref.) |  | 1.00 (ref.) |  |
| Yes | 0.95 (0.50–1.82) | 0.883 | 0.98 (0.29–3.31) | 0.971 | 1.41 (0.60–3.31) | 0.435 |
| ETP immunophenotype |  |  |  |  |  |  |
| Non-ETP | 1.00 (ref.) |  | 1.00 (ref.) |  | 1.00 (ref.) |  |
| ETP | 1.59 (0.81–3.11) | 0.175 | 2.85 (0.82–9.98) | 0.100 | 0.99 (0.40–2.41) | 0.975 |
| GVHD prophylaxis |  |  |  |  |  |  |
| CSA+MTX+MMF | 1.00 (ref.) |  | 1.00 (ref.) |  | 1.00 (ref.) |  |
| CSA+MTX+MMF+ATG | 1.38 (0.58–3.29) | 0.464 | 0.79 (0.18–3.59) | 0.765 | 0.24 (0.11–0.54) | <0.001 |
| MRD status before allo-HSCT |  |  |  |  |  |  |
| MRD+ | 1.00 (ref.) |  | 1.00 (ref.) |  | 1.00 (ref.) |  |
| MRD− | 1.24 (0.55–2.80) | 0.610 | 0.77 (0.17–3.61) | 0.745 | 0.43 (0.17–1.10) | 0.078 |
| Disease status at allo-HSCT |  |  |  |  |  |  |
| CR1 | 1.00 (ref.) |  | 1.00 (ref.) |  | 1.00 (ref.) |  |
| CR2 | 0.54 (0.14–2.07) | 0.373 | Not estimable | — | 0.58 (0.08–4.27) | 0.593 |
| Non-CR | 0.58 (0.18–1.87) | 0.358 | Not estimable | — | 0.93 (0.21–4.15) | 0.926 |
| HCT-CI |  |  |  |  |  |  |
| 0 | 1.00 (ref.) |  | 1.00 (ref.) |  | 1.00 (ref.) |  |
| 1 | 1.58 (0.82–3.05) | 0.175 | 1.46 (0.40–5.38) | 0.567 | 0.93 (0.32–2.75) | 0.902 |
| ≥2 | 1.95 (0.54–7.04) | 0.309 | 1.91 (0.24–15.11) | 0.541 | 2.06 (0.44–9.59) | 0.358 |
| Chemotherapy cycles before allo-HSCT |  |  |  |  |  |  |
| ≤4 | 1.00 (ref.) |  | 1.00 (ref.) |  | 1.00 (ref.) |  |
| ≥5 | 1.09 (0.56–2.11) | 0.800 | 0.29 (0.09–0.99) | 0.048 | 2.50 (0.86–7.29) | 0.094 |
| Donor type |  |  |  |  |  |  |
| MSD | 1.00 (ref.) |  | 1.00 (ref.) |  | 1.00 (ref.) |  |
| HID | 1.22 (0.61–2.45) | 0.573 | 0.44 (0.11–1.73) | 0.241 | 0.26 (0.11–0.64) | 0.003 |
| MUD | 1.28 (0.44–3.68) | 0.650 | 1.87 (0.42–8.31) | 0.410 | 0.15 (0.02–1.05) | 0.056 |
| Donor-recipient gender matching |  |  |  |  |  |  |
| Others | 1.00 (ref.) |  | 1.00 (ref.) |  | 1.00 (ref.) |  |
| Female-male | 1.44 (0.75–2.77) | 0.271 | 0.22 (0.03–1.75) | 0.153 | 1.19 (0.50–2.88) | 0.692 |
| Conditioning regimen |  |  |  |  |  |  |
| mBuCy | 1.00 (ref.) |  | 1.00 (ref.) |  | 1.00 (ref.) |  |
| TBI-Cy | Not estimable | — | Not estimable | — | Not estimable | — |
| Others | Not estimable | — | Not estimable | — | Not estimable | — |
| Time from diagnosis to allo-HSCT |  |  |  |  |  |  |
| <8 months | 1.00 (ref.) |  | 1.00 (ref.) |  | 1.00 (ref.) |  |
| ≥8 months | 0.93 (0.50–1.74) | 0.826 | 0.69 (0.21–2.32) | 0.550 | 1.46 (0.64–3.35) | 0.370 |
| Transplant era |  |  |  |  |  |  |
| 2010–2018 | 1.00 (ref.) |  | 1.00 (ref.) |  | 1.00 (ref.) |  |
| 2019–2025 | 1.82 (0.96–3.43) | 0.066 | 1.76 (0.52–5.99) | 0.363 | 0.60 (0.25–1.42) | 0.247 |
| MNC dose, per 1 ×10⁸/kg | 1.08 (0.99–1.17) | 0.094 | 0.96 (0.81–1.14) | 0.640 | 0.97 (0.88–1.07) | 0.578 |
| CD34+ cell dose, per 1 ×10⁶/kg | 0.98 (0.88–1.08) | 0.646 | 1.05 (0.93–1.20) | 0.423 | 0.96 (0.86–1.08) | 0.484 |

Each variable was examined separately using univariable Fine–Gray regression. Results are reported as sHRs with 95% confidence intervals. Reference categories are shown as 1.00 (ref.). Missing observations were excluded for the variable concerned. MRD was evaluated only among patients in complete remission with evaluable MRD. Not estimable indicates that a coefficient could not be estimated because of sparse or zero target events.

Abbreviations: aGVHD, acute graft-versus-host disease; NIH, National Institutes of Health; cGVHD, chronic graft-versus-host disease; sHR, subdistribution hazard ratio; CI, confidence interval; T-ALL, T-cell acute lymphoblastic leukemia; T-LBL, T-cell lymphoblastic lymphoma; allo-HSCT, allogeneic hematopoietic stem cell transplantation; BM, bone marrow; WBC, white blood cell; LDH, lactic dehydrogenase; CNS, central nervous system; ETP, early T-cell precursor; GVHD, graft-versus-host disease; CSA, cyclosporine A; MMF, mycophenolate mofetil; MTX, methotrexate; ATG, antithymocyte globulin; MRD, measurable residual disease; CR1, first complete remission; CR2, second complete remission; non-CR, non-complete remission; HCT-CI, hematopoietic cell transplantation comorbidity index; MSD, human leukocyte antigen (HLA)-matched sibling donors; HID, HLA-haploidentical related donors; MUD, HLA-matched unrelated donors; mBuCy, modified busulfan plus cyclophosphamide; TBI-Cy, total body irradiation plus cyclophosphamide; MNCs, mononuclear cells.

**Table S10. Univariable Schoenfeld residual tests for OS, DFS, and GRFS.**

| **Outcome** | **Variable** | **Schoenfeld test P value** |
| --- | --- | --- |
| OS | Sex | 0.597 |
|  | Diagnosis | 0.004 |
|  | Age at allo-HSCT | 0.154 |
|  | BM blasts at diagnosis | 0.003 |
|  | WBC at diagnosis | 0.727 |
|  | LDH at diagnosis | 0.695 |
|  | CNS involvement | 0.381 |
|  | Mediastinal involvement | 0.314 |
|  | ETP immunophenotype | 0.173 |
|  | GVHD prophylaxis | 0.896 |
|  | MRD status before allo-HSCT | 0.930 |
|  | Disease status at allo-HSCT | 0.798 |
|  | HCT-CI | 0.608 |
|  | Chemotherapy cycles before allo-HSCT | 0.362 |
|  | Donor type | 0.255 |
|  | Donor-recipient gender matching | 0.314 |
|  | Conditioning regimen | 0.586 |
|  | Time from diagnosis to allo-HSCT | 0.962 |
|  | Transplant era | 0.027 |
|  | MNC dose | 0.030 |
|  | CD34+ dose | 0.412 |
| DFS | Sex | 0.797 |
|  | Diagnosis | 0.003 |
|  | Age at allo-HSCT | 0.106 |
|  | BM blasts at diagnosis | 0.009 |
|  | WBC at diagnosis | 0.793 |
|  | LDH at diagnosis | 0.290 |
|  | CNS involvement | 0.626 |
|  | Mediastinal involvement | 0.458 |
|  | ETP immunophenotype | 0.052 |
|  | GVHD prophylaxis | 0.755 |
|  | MRD status before allo-HSCT | 0.419 |
|  | Disease status at allo-HSCT | 0.635 |
|  | HCT-CI | 0.819 |
|  | Chemotherapy cycles before allo-HSCT | 0.319 |
|  | Donor type | 0.947 |
|  | Donor-recipient gender matching | 0.944 |
|  | Conditioning regimen | 0.709 |
|  | Time from diagnosis to allo-HSCT | 0.269 |
|  | Transplant era | 0.606 |
|  | MNC dose | 0.227 |
|  | CD34+ dose | 0.820 |
| GRFS | Sex | 0.806 |
|  | Diagnosis | 0.193 |
|  | Age at allo-HSCT | 0.511 |
|  | BM blasts at diagnosis | 0.574 |
|  | WBC at diagnosis | 0.969 |
|  | LDH at diagnosis | 0.670 |
|  | CNS involvement | 0.796 |
|  | Mediastinal involvement | 0.873 |
|  | ETP immunophenotype | 0.802 |
|  | GVHD prophylaxis | 0.190 |
|  | MRD status before allo-HSCT | 0.428 |
|  | Disease status at allo-HSCT | 0.372 |
|  | HCT-CI | 0.693 |
|  | Chemotherapy cycles before allo-HSCT | 0.038 |
|  | Donor type | 0.706 |
|  | Donor-recipient gender matching | 0.290 |
|  | Conditioning regimen | 0.302 |
|  | Time from diagnosis to allo-HSCT | 0.352 |
|  | Transplant era | 0.816 |
|  | MNC dose | 0.107 |
|  | CD34+ dose | 0.710 |

Each test was based on a separate univariable Cox model. A P value below 0.05 indicated that the proportional hazards assumption was not met.

Abbreviations: OS, overall survival; DFS, disease-free survival; GRFS, graft-versus-host disease-free, relapse-free survival; allo-HSCT, allogeneic hematopoietic stem cell transplantation; BM, bone marrow; WBC, white blood cell count; LDH, lactic dehydrogenase; CNS, central nervous system; ETP, early T-cell precursor; GVHD, graft-versus-host disease; MRD, measurable residual disease; HCT-CI, hematopoietic cell transplantation comorbidity index; MNC, mononuclear cell.

**Table S11. Multivariable Schoenfeld residual tests for OS, DFS, and GRFS.**

| **Outcome** | **Tested term** | **Schoenfeld-test P value** |
| --- | --- | --- |
| OS | Diagnosis | 0.008 |
|  | Age at allo-HSCT, per 10 years | 0.341 |
|  | Sex | 0.670 |
|  | Disease status at allo-HSCT | 0.558 |
|  | HCT-CI, per category increase (0, 1, or ≥2) | 0.719 |
|  | Transplant era | 0.041 |
|  | GLOBAL | 0.080 |
| DFS | Diagnosis | 0.007 |
|  | Age at allo-HSCT, per 10 years | 0.197 |
|  | Sex | 0.755 |
|  | Disease status at allo-HSCT | 0.644 |
|  | HCT-CI, per category increase (0, 1, or ≥2) | 0.468 |
|  | Transplant era | 0.801 |
|  | GLOBAL | 0.183 |
| GRFS | Diagnosis | 0.350 |
|  | Age at allo-HSCT, per 10 years | 0.789 |
|  | Sex | 0.785 |
|  | Disease status at allo-HSCT | 0.192 |
|  | HCT-CI, per category increase (0, 1, or ≥2) | 0.621 |
|  | Transplant era | 0.845 |
|  | GLOBAL | 0.864 |

The covariates in each model were the same as those in the corresponding primary multivariable Cox model in Table 3. No additional variable selection was performed based on the univariable regression or Schoenfeld residual test results. GLOBAL indicates the test of the proportional hazards assumption for the multivariable Cox model as a whole.

Abbreviations: OS, overall survival; DFS, disease-free survival; GRFS, graft-versus-host disease-free, relapse-free survival; allo-HSCT, allogeneic hematopoietic stem cell transplantation; HCT-CI, hematopoietic cell transplantation comorbidity index.

**Table S12. Multivariable Schoenfeld residual tests for OS and DFS in patients in complete remission with evaluable MRD.**

| **Outcome** | **Tested term** | **Schoenfeld test P value** |
| --- | --- | --- |
| OS | Diagnosis | 0.017 |
|  | MRD status before allo-HSCT | 0.967 |
|  | Age at allo-HSCT, per 10 years | 0.947 |
|  | Sex | 0.419 |
|  | HCT-CI, per category increase (0, 1, or ≥2) | 0.989 |
|  | Transplant era | 0.031 |
|  | GLOBAL | 0.038 |
| DFS | Diagnosis | 0.015 |
|  | MRD status before allo-HSCT | 0.457 |
|  | Age at allo-HSCT, per 10 years | 0.754 |
|  | Sex | 0.959 |
|  | HCT-CI, per category increase (0, 1, or ≥2) | 0.249 |
|  | Transplant era | 0.770 |
|  | GLOBAL | 0.273 |

The same 94 patients and covariates as in the corresponding multivariable Cox models in Table S18 were analyzed. Disease status was not included because all patients were in complete remission. GLOBAL indicates the test of the proportional hazards assumption for the multivariable Cox model as a whole. Covariates that did not meet this assumption were further evaluated in Table S15.

Abbreviations: OS, overall survival; DFS, disease-free survival; MRD, measurable residual disease; allo-HSCT, allogeneic hematopoietic stem cell transplantation; HCT-CI, hematopoietic cell transplantation comorbidity index.

**Table S13. Univariable effects of covariates other than diagnosis over time.**

| **Outcome** | **Variable** | **Comparison or unit** | **Time point** | **HR(t) (95% CI)** | **P value at the specified time point** | **Interaction P value** |
| --- | --- | --- | --- | --- | --- | --- |
| OS | BM blasts at diagnosis | per 10% increase | 6 months | 0.87 (0.79–0.97) | 0.011 | 0.003 |
|  |  |  | 12 months | 0.99 (0.91–1.08) | 0.864 |  |
|  |  |  | 24 months | 1.14 (0.99–1.30) | 0.065 |  |
|  |  |  | 36 months | 1.23 (1.03–1.48) | 0.024 |  |
| OS | Transplant era | 2019–2025 (ref. 2010–2018) | 6 months | 0.61 (0.30–1.25) | 0.179 | 0.045 |
|  |  |  | 12 months | 1.04 (0.59–1.84) | 0.888 |  |
|  |  |  | 24 months | 1.82 (0.78–4.24) | 0.164 |  |
|  |  |  | 36 months | 2.55 (0.84–7.74) | 0.099 |  |
| OS | MNC dose | per 1 × 10^8^/kg increase | 6 months | 0.93 (0.83–1.03) | 0.163 | 0.040 |
|  |  |  | 12 months | 1.01 (0.93–1.09) | 0.869 |  |
|  |  |  | 24 months | 1.10 (0.98–1.23) | 0.122 |  |
|  |  |  | 36 months | 1.16 (0.99–1.35) | 0.073 |  |
| DFS | BM blasts at diagnosis | per 10% increase | 6 months | 0.94 (0.86–1.02) | 0.133 | 0.007 |
|  |  |  | 12 months | 1.04 (0.95–1.15) | 0.383 |  |
|  |  |  | 24 months | 1.17 (1.00–1.36) | 0.054 |  |
|  |  |  | 36 months | 1.25 (1.02–1.52) | 0.031 |  |
| GRFS | Chemotherapy cycles before allo-HSCT | ≥5 (ref. <5) | 6 months | 1.16 (0.69–1.95) | 0.566 | 0.058 |
|  |  |  | 12 months | 1.80 (0.85–3.83) | 0.127 |  |
|  |  |  | 24 months | 2.85 (0.90–9.04) | 0.075 |  |
|  |  |  | 36 months | 3.76 (0.91–15.49) | 0.066 |  |

The table includes covariates that did not meet the proportional hazards assumption in the univariable Schoenfeld residual tests reported in Table S10. Diagnosis is not included because the corresponding results are presented in Table 4.

Abbreviations: OS, overall survival; DFS, disease-free survival; GRFS, graft-versus-host disease-free, relapse-free survival; HR(t), hazard ratio at the specified time; CI, confidence interval; BM, bone marrow; MNCs, mononuclear cells; allo-HSCT, allogeneic hematopoietic stem cell transplantation.

**Table S14. Multivariable analyses of changes over time for OS, DFS, CIR, and NRM.**

| Outcome | Estimate type | Variable | Time point, months | Effect measure | HR, sHR, HR(t), or sHR(t) (95% CI) | P value | Term interaction P value | Joint interaction P value |
| --- | --- | --- | --- | --- | --- | --- | --- | --- |
| OS | Estimate at the specified time point | Diagnosis, T-LBL (ref. T-ALL) | 6 | HR(t) | 2.41 (1.10–5.28) | 0.028 | 0.011 | 0.008 |
|  |  | Diagnosis, T-LBL (ref. T-ALL) | 12 | HR(t) | 1.01 (0.50–2.03) | 0.984 |  |  |
|  |  | Diagnosis, T-LBL (ref. T-ALL) | 24 | HR(t) | 0.40 (0.13–1.27) | 0.120 |  |  |
|  |  | Diagnosis, T-LBL (ref. T-ALL) | 36 | HR(t) | 0.23 (0.05–1.06) | 0.059 |  |  |
|  |  | Transplant era, 2019–2025 (ref. 2010–2018) | 6 | HR(t) | 0.41 (0.18–0.92) | 0.031 | 0.090 |  |
|  |  | Transplant era, 2019–2025 (ref. 2010–2018) | 12 | HR(t) | 0.65 (0.33–1.26) | 0.200 |  |  |
|  |  | Transplant era, 2019–2025 (ref. 2010–2018) | 24 | HR(t) | 1.05 (0.42–2.61) | 0.920 |  |  |
|  |  | Transplant era, 2019–2025 (ref. 2010–2018) | 36 | HR(t) | 1.40 (0.44–4.50) | 0.571 |  |  |
|  | Other model covariate | Age at allo-HSCT, per 10 years |  | HR | 1.08 (0.81–1.45) | 0.596 |  |  |
|  |  | Sex, female (ref. male) |  | HR | 0.40 (0.19–0.84) | 0.016 |  |  |
|  |  | Disease status at allo-HSCT, non-CR (ref. CR) |  | HR | 1.12 (0.45–2.77) | 0.803 |  |  |
|  |  | HCT-CI, per category increase (0, 1, or ≥2) |  | HR | 1.79 (1.06–3.02) | 0.029 |  |  |
| DFS | Estimate at the specified time point | Diagnosis, T-LBL (ref. T-ALL) | 6 | HR(t) | 1.58 (0.82–3.03) | 0.170 | 0.009 | 0.009 |
|  |  | Diagnosis, T-LBL (ref. T-ALL) | 12 | HR(t) | 0.67 (0.29–1.55) | 0.347 |  |  |
|  |  | Diagnosis, T-LBL (ref. T-ALL) | 24 | HR(t) | 0.27 (0.07–1.08) | 0.064 |  |  |
|  |  | Diagnosis, T-LBL (ref. T-ALL) | 36 | HR(t) | 0.16 (0.03–0.91) | 0.039 |  |  |
|  | Other model covariate | Age at allo-HSCT, per 10 years |  | HR | 1.13 (0.86–1.47) | 0.383 |  |  |
|  |  | Sex, female (ref. male) |  | HR | 0.48 (0.24–0.95) | 0.037 |  |  |
|  |  | Disease status at allo-HSCT, non-CR (ref. CR) |  | HR | 1.12 (0.46–2.76) | 0.801 |  |  |
|  |  | HCT-CI, per category increase (0, 1, or ≥2) |  | HR | 1.70 (1.03–2.81) | 0.038 |  |  |
|  |  | Transplant era, 2019–2025 (ref. 2010–2018) |  | HR | 0.69 (0.37–1.29) | 0.247 |  |  |
| CIR | Estimate at the specified time point | Diagnosis, T-LBL (ref. T-ALL) | 6 | sHR(t) | 1.16 (0.56–2.41) | 0.689 | 0.032 | 0.032 |
|  |  | Diagnosis, T-LBL (ref. T-ALL) | 12 | sHR(t) | 0.52 (0.19–1.43) | 0.203 |  |  |
|  |  | Diagnosis, T-LBL (ref. T-ALL) | 24 | sHR(t) | 0.22 (0.04–1.15) | 0.073 |  |  |
|  |  | Diagnosis, T-LBL (ref. T-ALL) | 36 | sHR(t) | 0.13 (0.02–1.06) | 0.057 |  |  |
|  | Other model covariate | Age at allo-HSCT, per 10 years |  | sHR | 0.99 (0.73–1.34) | 0.954 |  |  |
|  |  | Disease status at allo-HSCT, non-CR (ref. CR) |  | sHR | 2.07 (0.92–4.63) | 0.078 |  |  |
|  |  | HCT-CI, per category increase (0, 1, or ≥2) |  | sHR | 1.69 (0.98–2.93) | 0.061 |  |  |
|  |  | Transplant era, 2019–2025 (ref. 2010–2018) |  | sHR | 1.32 (0.68–2.55) | 0.415 |  |  |
| NRM | Estimate at the specified time point | Diagnosis, T-LBL (ref. T-ALL) | 6 | sHR(t) | 2.06 (0.63–6.75) | 0.234 | 0.008 | 0.008 |
|  |  | Diagnosis, T-LBL (ref. T-ALL) | 12 | sHR(t) | 0.56 (0.16–1.96) | 0.368 |  |  |
|  |  | Diagnosis, T-LBL (ref. T-ALL) | 24 | sHR(t) | 0.14 (0.02–0.98) | 0.047 |  |  |
|  |  | Diagnosis, T-LBL (ref. T-ALL) | 36 | sHR(t) | 0.06 (0.01–0.72) | 0.026 |  |  |
|  | Other model covariate | HCT-CI, per category increase (0, 1, or ≥2) |  | sHR | 1.11 (0.36–3.36) | 0.860 |  |  |
|  |  | Transplant era, 2019–2025 (ref. 2010–2018) |  | sHR | 0.47 (0.13–1.74) | 0.259 |  |  |

The covariates in each model were the same as those in the corresponding primary multivariable model in Table 3. The OS Cox model included time interactions for diagnosis and transplant era. The DFS Cox model and the CIR and NRM Fine–Gray models included a time interaction for diagnosis. The remaining covariates were retained without time interactions. The term interaction P value assesses the time interaction for the corresponding variable. The joint interaction P value assesses all time interactions in the model together. When a model contained only one time interaction, the term and joint interaction P values were identical. HR(t) and sHR(t) are reported at 6, 12, 24, and 36 months.

Abbreviations: OS, overall survival; DFS, disease-free survival; CIR, cumulative incidence of relapse; NRM, non-relapse mortality; HR, hazard ratio; HR(t), hazard ratio at the specified time; sHR, subdistribution hazard ratio; sHR(t), subdistribution hazard ratio at the specified time; CI, confidence interval; T-ALL, T-cell acute lymphoblastic leukemia; T-LBL, T-cell lymphoblastic lymphoma; allo-HSCT, allogeneic hematopoietic stem cell transplantation; CR, complete remission; non-CR, non-complete remission; HCT-CI, hematopoietic cell transplantation comorbidity index.

**Table S15. Multivariable effects over time for OS and DFS in patients in complete remission with evaluable MRD.**

| **Outcome** | **Variable** | **Time point** | **HR(t) (95% CI)** | **P value at the specified time point** | **Interaction P value** | **Joint interaction P value** |
| --- | --- | --- | --- | --- | --- | --- |
| OS | Diagnosis, T-LBL (ref. T-ALL) | 6 months | 2.50 (1.07–5.85) | 0.035 | 0.013 | 0.008 |
|  |  | 12 months | 0.90 (0.40–2.02) | 0.797 |  |  |
|  |  | 24 months | 0.31 (0.07–1.26) | 0.101 |  |  |
|  |  | 36 months | 0.16 (0.02–1.04) | 0.055 |  |  |
| OS | Transplant era, 2019–2025 (ref. 2010–2018) | 6 months | 0.38 (0.16–0.93) | 0.033 | 0.054 | 0.008 |
|  |  | 12 months | 0.68 (0.33–1.40) | 0.299 |  |  |
|  |  | 24 months | 1.26 (0.47–3.41) | 0.647 |  |  |
|  |  | 36 months | 1.82 (0.50–6.57) | 0.360 |  |  |
| DFS | Diagnosis, T-LBL (ref. T-ALL) | 6 months | 1.58 (0.77–3.22) | 0.212 | 0.016 | 0.016 |
|  |  | 12 months | 0.62 (0.23–1.68) | 0.343 |  |  |
|  |  | 24 months | 0.23 (0.04–1.22) | 0.084 |  |  |
|  |  | 36 months | 0.13 (0.02–1.05) | 0.056 |  |  |

The models used the same 94 patients and covariates as those in Table S18. Table S15 reports time specific HRs for variables that did not meet the proportional hazards assumption in Table S12. Time interactions were included for diagnosis and transplant era in the OS model and for diagnosis in the DFS model. Table S18 presents one adjusted HR for each covariate from models that assumed the HR remained constant during follow-up. MRD met the proportional hazards assumption and was not evaluated using a time interaction.

Abbreviations: OS, overall survival; DFS, disease-free survival; MRD, measurable residual disease; CR, complete remission; HR(t), hazard ratio at the specified time; CI, confidence interval; T-ALL, T-cell acute lymphoblastic leukemia; T-LBL, T-cell lymphoblastic lymphoma.

**Table S16. Tests of changes over time in multivariable Fine–Gray models for GVHD outcomes.**

| **Outcome** | **Model covariates** | **Tested term** | **Interaction**  **P value** | **N** | **GVHD events** | **Competing events** |
| --- | --- | --- | --- | --- | --- | --- |
| Grade II–IV aGVHD | Diagnosis, age at allo-HSCT per 10 years, and transplant era | Diagnosis | 0.162 | 106 | 39 | 7 |
|  |  | Age at allo-HSCT, per 10 years | 0.296 |  |  |  |
|  |  | Transplant era, 2019–2025 (ref. 2010–2018) | 0.811 |  |  |  |
| Any NIH cGVHD | Diagnosis and donor type | Diagnosis | 0.467 | 106 | 21 | 47 |
|  |  | Donor type, overall | Not estimable |  |  |  |

All covariates listed under Model covariates were retained in each model, and Tested term identifies the covariate whose interaction with log(1+time) was assessed. The interaction P value indicates whether the corresponding sHR changed during follow-up. For donor type, the overall interaction test assessed the variable as a whole, but the interaction effect could not be estimated in the any NIH cGVHD model because the model did not converge.

Abbreviations: GVHD, graft-versus-host disease; aGVHD, acute graft-versus-host disease; NIH, National Institutes of Health; cGVHD, chronic graft-versus-host disease; allo-HSCT, allogeneic hematopoietic stem cell transplantation.

**Table S17. Complete case sensitivity analyses involving WBC and LDH for OS, DFS, CIR, and GRFS.**

| **Outcome** | **Complete case sample** | **Model** | **Variable** | **Effect measure** | **HR or sHR (95% CI)** | **P value** |
| --- | --- | --- | --- | --- | --- | --- |
| OS | WBC complete cases | Model without WBC | Diagnosis, T-LBL (ref. T-ALL) | HR | 1.33 (0.73–2.45) | 0.354 |
|  |  |  | Age at allo-HSCT, per 10 years |  | 1.12 (0.82–1.51) | 0.482 |
|  |  |  | Sex, female (ref. male) |  | 0.38 (0.17–0.83) | 0.015 |
|  |  |  | Disease status, non-CR (ref. CR) |  | 1.07 (0.41–2.81) | 0.890 |
|  |  |  | HCT-CI, per category increase (0, 1, or ≥2) |  | 1.77 (1.01–3.11) | 0.045 |
|  |  |  | Transplant era, 2019–2025 (ref. 2010–2018) |  | 0.57 (0.29–1.11) | 0.097 |
|  | WBC complete cases | Model including WBC | Diagnosis, T-LBL (ref. T-ALL) | HR | 1.30 (0.70–2.41) | 0.402 |
|  |  |  | Age at allo-HSCT, per 10 years |  | 1.12 (0.83–1.52) | 0.457 |
|  |  |  | Sex, female (ref. male) |  | 0.38 (0.17–0.83) | 0.015 |
|  |  |  | Disease status, non-CR (ref. CR) |  | 1.06 (0.40–2.78) | 0.905 |
|  |  |  | HCT-CI, per category increase (0, 1, or ≥2) |  | 1.77 (1.01–3.10) | 0.044 |
|  |  |  | Transplant era, 2019–2025 (ref. 2010–2018) |  | 0.57 (0.29–1.11) | 0.096 |
|  |  |  | WBC at diagnosis, >100 × 10^9^/L (ref. ≤100 × 10^9^/L) |  | 0.79 (0.24–2.63) | 0.702 |
|  | LDH complete cases | Model without LDH | Diagnosis, T-LBL (ref. T-ALL) | HR | 1.65 (0.88–3.10) | 0.117 |
|  |  |  | Age at allo-HSCT, per 10 years |  | 1.07 (0.79–1.44) | 0.670 |
|  |  |  | Sex, female (ref. male) |  | 0.45 (0.21–0.97) | 0.040 |
|  |  |  | Disease status, non-CR (ref. CR) |  | 0.96 (0.36–2.57) | 0.942 |
|  |  |  | HCT-CI, per category increase (0, 1, or ≥2) |  | 1.90 (1.11–3.25) | 0.020 |
|  |  |  | Transplant era, 2019–2025 (ref. 2010–2018) |  | 0.71 (0.35–1.43) | 0.336 |
|  | LDH complete cases | Model including LDH | Diagnosis, T-LBL (ref. T-ALL) | HR | 1.67 (0.89–3.14) | 0.110 |
|  |  |  | Age at allo-HSCT, per 10 years |  | 1.08 (0.79–1.48) | 0.618 |
|  |  |  | Sex, female (ref. male) |  | 0.47 (0.21–1.03) | 0.059 |
|  |  |  | Disease status, non-CR (ref. CR) |  | 0.96 (0.36–2.55) | 0.928 |
|  |  |  | HCT-CI, per category increase (0, 1, or ≥2) |  | 1.87 (1.09–3.22) | 0.023 |
|  |  |  | Transplant era, 2019–2025 (ref. 2010–2018) |  | 0.71 (0.35–1.44) | 0.346 |
|  |  |  | LDH at diagnosis, elevated (ref. normal) |  | 1.12 (0.60–2.10) | 0.723 |
|  | WBC+LDH complete cases | Model without WBC or LDH | Diagnosis, T-LBL (ref. T-ALL) | HR | 1.63 (0.86–3.06) | 0.132 |
|  |  |  | Age at allo-HSCT, per 10 years |  | 1.05 (0.77–1.43) | 0.773 |
|  |  |  | Sex, female (ref. male) |  | 0.43 (0.19–0.95) | 0.037 |
|  |  |  | Disease status, non-CR (ref. CR) |  | 0.88 (0.30–2.56) | 0.813 |
|  |  |  | HCT-CI, per category increase (0, 1, or ≥2) |  | 1.83 (1.06–3.16) | 0.030 |
|  |  |  | Transplant era, 2019–2025 (ref. 2010–2018) |  | 0.73 (0.36–1.48) | 0.385 |
|  | WBC+LDH complete cases | Model including WBC and LDH | Diagnosis, T-LBL (ref. T-ALL) | HR | 1.60 (0.84–3.04) | 0.153 |
|  |  |  | Age at allo-HSCT, per 10 years |  | 1.07 (0.78–1.48) | 0.671 |
|  |  |  | Sex, female (ref. male) |  | 0.45 (0.20–1.02) | 0.057 |
|  |  |  | Disease status, non-CR (ref. CR) |  | 0.86 (0.29–2.49) | 0.776 |
|  |  |  | HCT-CI, per category increase (0, 1, or ≥2) |  | 1.80 (1.04–3.10) | 0.035 |
|  |  |  | Transplant era, 2019–2025 (ref. 2010–2018) |  | 0.74 (0.36–1.51) | 0.403 |
|  |  |  | WBC at diagnosis, >100 × 10^9^/L (ref. ≤100 × 10^9^/L) |  | 0.76 (0.22–2.56) | 0.655 |
|  |  |  | LDH at diagnosis, elevated (ref. normal) |  | 1.15 (0.61–2.18) | 0.669 |
| DFS | WBC complete cases | Model without WBC | Diagnosis, T-LBL (ref. T-ALL) | HR | 1.24 (0.68–2.26) | 0.483 |
|  |  |  | Age at allo-HSCT, per 10 years |  | 1.13 (0.85–1.50) | 0.396 |
|  |  |  | Sex, female (ref. male) |  | 0.46 (0.23–0.95) | 0.035 |
|  |  |  | Disease status, non-CR (ref. CR) |  | 1.06 (0.41–2.76) | 0.899 |
|  |  |  | HCT-CI, per category increase (0, 1, or ≥2) |  | 1.84 (1.07–3.18) | 0.029 |
|  |  |  | Transplant era, 2019–2025 (ref. 2010–2018) |  | 0.63 (0.34–1.20) | 0.161 |
|  | WBC complete cases | Model including WBC | Diagnosis, T-LBL (ref. T-ALL) | HR | 1.25 (0.68–2.30) | 0.479 |
|  |  |  | Age at allo-HSCT, per 10 years |  | 1.13 (0.85–1.50) | 0.404 |
|  |  |  | Sex, female (ref. male) |  | 0.46 (0.23–0.95) | 0.035 |
|  |  |  | Disease status, non-CR (ref. CR) |  | 1.07 (0.41–2.77) | 0.895 |
|  |  |  | HCT-CI, per category increase (0, 1, or ≥2) |  | 1.84 (1.07–3.19) | 0.029 |
|  |  |  | Transplant era, 2019–2025 (ref. 2010–2018) |  | 0.63 (0.34–1.20) | 0.161 |
|  |  |  | WBC at diagnosis, >100 × 10^9^/L (ref. ≤100 × 10^9^/L) |  | 1.05 (0.37–3.03) | 0.922 |
|  | LDH complete cases | Model without LDH | Diagnosis, T-LBL (ref. T-ALL) | HR | 1.51 (0.82–2.80) | 0.189 |
|  |  |  | Age at allo-HSCT, per 10 years |  | 1.06 (0.81–1.41) | 0.658 |
|  |  |  | Sex, female (ref. male) |  | 0.53 (0.26–1.08) | 0.080 |
|  |  |  | Disease status, non-CR (ref. CR) |  | 0.93 (0.35–2.45) | 0.884 |
|  |  |  | HCT-CI, per category increase (0, 1, or ≥2) |  | 1.94 (1.15–3.28) | 0.014 |
|  |  |  | Transplant era, 2019–2025 (ref. 2010–2018) |  | 0.83 (0.42–1.62) | 0.586 |
|  | LDH complete cases | Model including LDH | Diagnosis, T-LBL (ref. T-ALL) | HR | 1.57 (0.84–2.92) | 0.154 |
|  |  |  | Age at allo-HSCT, per 10 years |  | 1.11 (0.83–1.49) | 0.465 |
|  |  |  | Sex, female (ref. male) |  | 0.59 (0.28–1.23) | 0.157 |
|  |  |  | Disease status, non-CR (ref. CR) |  | 0.89 (0.34–2.35) | 0.821 |
|  |  |  | HCT-CI, per category increase (0, 1, or ≥2) |  | 1.89 (1.12–3.19) | 0.017 |
|  |  |  | Transplant era, 2019–2025 (ref. 2010–2018) |  | 0.84 (0.43–1.65) | 0.618 |
|  |  |  | LDH at diagnosis, elevated (ref. normal) |  | 1.38 (0.74–2.58) | 0.305 |
|  | WBC+LDH complete cases | Model without WBC or LDH | Diagnosis, T-LBL (ref. T-ALL) | HR | 1.50 (0.80–2.79) | 0.205 |
|  |  |  | Age at allo-HSCT, per 10 years |  | 1.06 (0.79–1.41) | 0.713 |
|  |  |  | Sex, female (ref. male) |  | 0.52 (0.25–1.08) | 0.082 |
|  |  |  | Disease status, non-CR (ref. CR) |  | 0.89 (0.31–2.55) | 0.832 |
|  |  |  | HCT-CI, per category increase (0, 1, or ≥2) |  | 1.91 (1.12–3.25) | 0.017 |
|  |  |  | Transplant era, 2019–2025 (ref. 2010–2018) |  | 0.84 (0.43–1.66) | 0.619 |
|  | WBC+LDH complete cases | Model including WBC and LDH | Diagnosis, T-LBL (ref. T-ALL) | HR | 1.54 (0.82–2.90) | 0.182 |
|  |  |  | Age at allo-HSCT, per 10 years |  | 1.11 (0.82–1.50) | 0.503 |
|  |  |  | Sex, female (ref. male) |  | 0.58 (0.28–1.23) | 0.159 |
|  |  |  | Disease status, non-CR (ref. CR) |  | 0.85 (0.30–2.43) | 0.757 |
|  |  |  | HCT-CI, per category increase (0, 1, or ≥2) |  | 1.86 (1.09–3.15) | 0.022 |
|  |  |  | Transplant era, 2019–2025 (ref. 2010–2018) |  | 0.86 (0.43–1.70) | 0.660 |
|  |  |  | WBC at diagnosis, >100 × 10^9^/L (ref. ≤100 × 10^9^/L) |  | 0.93 (0.32–2.72) | 0.893 |
|  |  |  | LDH at diagnosis, elevated (ref. normal) |  | 1.40 (0.74–2.64) | 0.296 |
| CIR | LDH complete cases | Model without LDH | Diagnosis, T-LBL (ref. T-ALL) | sHR | 1.11 (0.53–2.33) | 0.784 |
|  |  |  | Age at allo-HSCT, per 10 years |  | 0.94 (0.69–1.27) | 0.691 |
|  |  |  | Disease status, non-CR (ref. CR) |  | 2.09 (0.91–4.81) | 0.082 |
|  |  |  | HCT-CI, per category increase (0, 1, or ≥2) |  | 1.72 (0.98–3.01) | 0.059 |
|  |  |  | Transplant era, 2019–2025 (ref. 2010–2018) |  | 1.78 (0.85–3.75) | 0.127 |
|  | LDH complete cases | Model including LDH | Diagnosis, T-LBL (ref. T-ALL) | sHR | 1.26 (0.59–2.68) | 0.545 |
|  |  |  | Age at allo-HSCT, per 10 years |  | 1.07 (0.77–1.49) | 0.700 |
|  |  |  | Disease status, non-CR (ref. CR) |  | 1.75 (0.77–3.98) | 0.182 |
|  |  |  | HCT-CI, per category increase (0, 1, or ≥2) |  | 1.68 (1.01–2.79) | 0.046 |
|  |  |  | Transplant era, 2019–2025 (ref. 2010–2018) |  | 1.62 (0.75–3.49) | 0.216 |
|  |  |  | LDH at diagnosis, elevated (ref. normal) |  | 2.35 (1.09–5.09) | 0.029 |
| GRFS | WBC complete cases | Model without WBC | Diagnosis, T-LBL (ref. T-ALL) | HR | 0.68 (0.39–1.19) | 0.175 |
|  |  |  | Age at allo-HSCT, per 10 years |  | 1.07 (0.84–1.36) | 0.601 |
|  |  |  | Sex, female (ref. male) |  | 0.58 (0.32–1.03) | 0.063 |
|  |  |  | Disease status, non-CR (ref. CR) |  | 1.16 (0.52–2.61) | 0.720 |
|  |  |  | HCT-CI, per category increase (0, 1, or ≥2) |  | 2.48 (1.53–4.03) | <0.001 |
|  |  |  | Transplant era, 2019-2025 (ref. 2010-2018) |  | 0.54 (0.31–0.95) | 0.032 |
|  | WBC complete cases | Model including WBC | Diagnosis, T-LBL (ref. T-ALL) | HR | 0.67 (0.38–1.17) | 0.160 |
|  |  |  | Age at allo-HSCT, per 10 years |  | 1.07 (0.84–1.36) | 0.588 |
|  |  |  | Sex, female (ref. male) |  | 0.58 (0.32–1.04) | 0.066 |
|  |  |  | Disease status, non-CR (ref. CR) |  | 1.15 (0.51–2.58) | 0.735 |
|  |  |  | HCT-CI, per category increase (0, 1, or ≥2) |  | 2.49 (1.54–4.04) | <0.001 |
|  |  |  | Transplant era, 2019-2025 (ref. 2010-2018) |  | 0.55 (0.31–0.96) | 0.035 |
|  |  |  | WBC at diagnosis, >100 × 10^9^/L (ref. ≤100 × 10^9^/L) |  | 0.83 (0.32–2.13) | 0.700 |
|  | LDH complete cases | Model without LDH | Diagnosis, T-LBL (ref. T-ALL) | HR | 0.79 (0.44–1.41) | 0.419 |
|  |  |  | Age at allo-HSCT, per 10 years |  | 1.06 (0.84–1.35) | 0.621 |
|  |  |  | Sex, female (ref. male) |  | 0.64 (0.35–1.15) | 0.137 |
|  |  |  | Disease status, non-CR (ref. CR) |  | 1.18 (0.53–2.67) | 0.684 |
|  |  |  | HCT-CI, per category increase (0, 1, or ≥2) |  | 2.51 (1.56–4.05) | <0.001 |
|  |  |  | Transplant era, 2019-2025 (ref. 2010-2018) |  | 0.63 (0.34–1.15) | 0.132 |
|  | LDH complete cases | Model including LDH | Diagnosis, T-LBL (ref. T-ALL) | HR | 0.77 (0.42–1.40) | 0.391 |
|  |  |  | Age at allo-HSCT, per 10 years |  | 1.05 (0.82–1.35) | 0.693 |
|  |  |  | Sex, female (ref. male) |  | 0.61 (0.33–1.15) | 0.127 |
|  |  |  | Disease status, non-CR (ref. CR) |  | 1.20 (0.53–2.71) | 0.662 |
|  |  |  | HCT-CI, per category increase (0, 1, or ≥2) |  | 2.56 (1.57–4.18) | <0.001 |
|  |  |  | Transplant era, 2019-2025 (ref. 2010-2018) |  | 0.62 (0.34–1.14) | 0.126 |
|  |  |  | LDH at diagnosis, elevated (ref. normal) |  | 0.90 (0.52–1.56) | 0.707 |
|  | WBC+LDH complete cases | Model without WBC or LDH | Diagnosis, T-LBL (ref. T-ALL) | HR | 0.77 (0.43–1.40) | 0.395 |
|  |  |  | Age at allo-HSCT, per 10 years |  | 1.04 (0.82–1.34) | 0.728 |
|  |  |  | Sex, female (ref. male) |  | 0.62 (0.34–1.13) | 0.117 |
|  |  |  | Disease status, non-CR (ref. CR) |  | 1.10 (0.46–2.60) | 0.833 |
|  |  |  | HCT-CI, per category increase (0, 1, or ≥2) |  | 2.46 (1.52–3.98) | <0.001 |
|  |  |  | Transplant era, 2019-2025 (ref. 2010-2018) |  | 0.64 (0.35–1.17) | 0.149 |
|  | WBC+LDH complete cases | Model including WBC and LDH | Diagnosis, T-LBL (ref. T-ALL) | HR | 0.75 (0.41–1.37) | 0.347 |
|  |  |  | Age at allo-HSCT, per 10 years |  | 1.04 (0.81–1.34) | 0.766 |
|  |  |  | Sex, female (ref. male) |  | 0.61 (0.32–1.16) | 0.128 |
|  |  |  | Disease status, non-CR (ref. CR) |  | 1.10 (0.46–2.61) | 0.837 |
|  |  |  | HCT-CI, per category increase (0, 1, or ≥2) |  | 2.50 (1.53–4.09) | <0.001 |
|  |  |  | Transplant era, 2019-2025 (ref. 2010-2018) |  | 0.65 (0.35–1.20) | 0.164 |
|  |  |  | WBC at diagnosis, >100 × 10^9^/L (ref. ≤100 × 10^9^/L) |  | 0.81 (0.30–2.14) | 0.665 |
|  |  |  | LDH at diagnosis, elevated (ref. normal) |  | 0.94 (0.53–1.66) | 0.824 |

The complete case samples included 104 patients for WBC, 92 patients for LDH, and 91 patients for the joint WBC and LDH analysis.

Abbreviations: WBC, white blood cell count; LDH, lactic dehydrogenase; OS, overall survival; DFS, disease-free survival; CIR, cumulative incidence of relapse; GRFS, graft-versus-host disease–free, relapse-free survival; HR, hazard ratio; sHR, subdistribution hazard ratio; CI, confidence interval; T-ALL, T-cell acute lymphoblastic leukemia; T-LBL, T-cell lymphoblastic lymphoma; allo-HSCT, allogeneic hematopoietic stem-cell transplantation; CR, complete remission; non-CR, non-complete remission; HCT-CI, hematopoietic cell transplantation comorbidity index.

**Table S18. Multivariable analyses for OS and DFS in patients in complete remission with evaluable MRD.**

| **Outcome** | **Variable** | **HR (95% CI)** | **P value** |
| --- | --- | --- | --- |
| OS | Diagnosis, T-LBL (ref. T-ALL) | 1.37 (0.71–2.63) | 0.344 |
|  | MRD status before allo-HSCT, negative (ref. positive) | 0.85 (0.40–1.82) | 0.678 |
|  | Age at allo-HSCT, per 10 years | 1.14 (0.83–1.58) | 0.417 |
|  | Sex, female (ref. male) | 0.45 (0.20–0.99) | 0.048 |
|  | HCT-CI, per category increase (0, 1, or ≥2) | 1.75 (1.01–3.03) | 0.045 |
|  | Transplant era, 2019–2025 (ref. 2010–2018) | 0.56 (0.28–1.14) | 0.111 |
| DFS | Diagnosis, T-LBL (ref. T-ALL) | 1.28 (0.67–2.44) | 0.456 |
|  | MRD status before allo-HSCT, negative (ref. positive) | 0.80 (0.38–1.66) | 0.548 |
|  | Age at allo-HSCT, per 10 years | 1.14 (0.85–1.53) | 0.387 |
|  | Sex, female (ref. male) | 0.57 (0.27–1.19) | 0.133 |
|  | HCT-CI, per category increase (0, 1, or ≥2) | 1.80 (1.06–3.05) | 0.030 |
|  | Transplant era, 2019–2025 (ref. 2010–2018) | 0.66 (0.34–1.30) | 0.233 |

The analysis included 94 patients who were in complete remission at allo-HSCT and had evaluable pretransplant MRD results. Each model included the same covariates as the corresponding primary multivariable model in Table 3, except that disease status was omitted because it did not vary within this subgroup and MRD status was added.

Abbreviations: OS, overall survival; DFS, disease-free survival; MRD, measurable residual disease; HR, hazard ratio; CI, confidence interval; T-ALL, T-cell acute lymphoblastic leukemia; T-LBL, T-cell lymphoblastic lymphoma; allo-HSCT, allogeneic hematopoietic stem cell transplantation; HCT-CI, hematopoietic cell transplantation comorbidity index.
